# miR-122-SOCS1-JAK2 axis regulates allergic inflammation and allergic inflammation-promoted cellular interactions

**DOI:** 10.18632/oncotarget.19149

**Published:** 2017-07-10

**Authors:** Kyeonga Noh, Misun Kim, Youngmi Kim, Hanearl Kim, Hyuna Kim, Jaehwan Byun, Yeongseo Park, Hansoo Lee, Yun Sil Lee, Jongseon Choe, Young Myeong Kim, Dooil Jeoung

**Affiliations:** ^1^ Department of Biochemistry, Kangwon National University, Chunchon 24341, Korea; ^2^ Department of Biological Sciences, Kangwon National University, Chunchon 24341, Korea; ^3^ College of Pharmacy, Ewha Womans University, Seoul 03760, Korea; ^4^ Graduate School of Medicine, Kangwon National University, Chunchon 24341, Korea

**Keywords:** allergic inflammation, cellular interaction, miR-122, SOCS1, tumor microenvironments

## Abstract

The regulatory role of suppressor of cytokine signaling 1 (SOCS1) in inflammation has been reported. However, its role in allergic inflammation has not been previously reported. SOCS1 mediated *in vitro* and *in vivo* allergic inflammation. Histone deacetylase-3 (HDAC3), a mediator of allergic inflammation, interacted with SOCS1, and miR-384 inhibitor, a positive regulator of HDAC3, induced features of allergic inflammation in an SOCS1-dependent manner. miRNA array analysis showed that the expression of miR-122 was decreased by antigen-stimulation. TargetScan analysis predicted the binding of miR-122 to the 3′-UTR of SOCS1. miR-122 inhibitor induced *in vitro* and *in vivo* allergic features in SOCS1-dependent manner. SOCS1 was necessary for allergic inflammation-promoted enhanced tumorigenic and metastatic potential of cancer cells. SOCS1 and miR-122 regulated cellular interactions involving cancer cells, mast cells and macrophages during allergic inflammation. SOCS1 mimetic peptide, D-T-H-F-R-T-F-R-S-H-S-D-Y-R-R-I, inhibited *in vitro* and *in vivo* allergic inflammation, allergic inflammation-promoted enhanced tumorigenic and metastatic potential of cancer cells, and cellular interactions during allergic inflammation. Janus kinase 2 (JAK2) exhibited binding to SOCS1 mimetic peptide and mediated allergic inflammation. Transforming growth factor- Δ1 (TGF-Δ1) was decreased during allergic inflammation and showed an anti-allergic effect. SOCS1 and JAK2 regulated the production of anti-allergic TGF-Δ1. Taken together, our results show that miR-122-SOCS1 feedback loop can be employed as a target for the development of anti-allergic and anti-cancer drugs.

## INTRODUCTION

SOCS1 inhibits excessive cytokine signaling, based on the fact that SOCS1-knockout (KO) mice die of severe inflammation within three weeks of birth [1]. SOCS1 decreases IL-6 production in LPS-stimulated RAW 264.7 cells [2]. Secretory SOCS1 exerts anti-inflammatory effects [3]. Neuroprotective and anti-inflamamtory effects of resveratrol result from the induction of SOCS1 [4]. Carvedilol reduces oxidative stress, inflammatory response and fibrosis in ethanol-induced liver injury in a rat model by induction of SOCS1 and suppression of inflammatory cytokines [5]. Olmesartan exerts an anti-inflammatory effect by upregulating SOCS1 [6]. Goat whey exerts a preventive effect against intestinal damage induced by acetic acid by upregulating SOCS1 [7].

miR-19promotes Th2 cytokine production by targeting SOCS1 [8]. miR-155 enhances inflammatory response in atherosclerosis by increasing STAT3 and NF-κB signaling via targeting SOCS1 [9]. miR-155 exerts an antiangiogenic but proarteriogenic function in the regulation of neovascularization via the suppression of SOCS1 [10]. The down-regulation miR-155 leads to anti-inflammatory effects by upregulating the expression of SOCS1 [11]. miR-155 promotes ulcerative colitis by targeting SOCS1 [12]. These reports indicate the role of SOCS1 as an anti-inflammatory molecule.

SOCS1 also plays a regulatory role in anaphylactic shock viscera injury processes [13]. SOCS1 promotes TGF-β-induced COX-2 expression and prostaglandin (PG) production by facilitating Smad3 phosphorylation and Snail binding to the COX-2 promoter [14]. The role of COX-2 in allergic inflammation and cellular interaction during allergic inflammation has been reported [15]. The expression of SOCS1 is increased in asthmatic bronchial epithelium [16]. SOCS1-mimetic peptides inhibit ocular inflammatory diseases including scleritis [17]. AT-RvD1, lipid mediator of inflammation resolution, ameliorates some of the most important phenotypes of experimental asthma by decreasing the expression of SOCS1 [18]. These reports imply the role of SOCS1 in allergic inflammation.

Many reports have indicated contradictory roles of SOCS1 in inflammation. The role of SOCS1 in allergic inflammation, such as anaphylaxis has not been investigated. The molecular network involving SOCS1 remains largely unknown. In this study, we present evidence that miR-122-SOCS1 negative feedback loop regulates allergic inflammation and cellular interactions during allergic inflammation-promoted enhanced tumorigenic and metastatic potential. SOCS1 mimetic peptide inhibits allergic inflammation and cellular interactions by allergic inflammation. We also report the role of JAK2 in allergic inflammation regulated by miR-122-SOCS1 feedback loop. The role of miR-122-SOCS1-JAK2 loop in allergic inflammation, such as anaphylaxis, has not been previously reported.

## RESULTS

### SOCS1 mediates *in vitro* allergic inflammation

**Figure 1:**
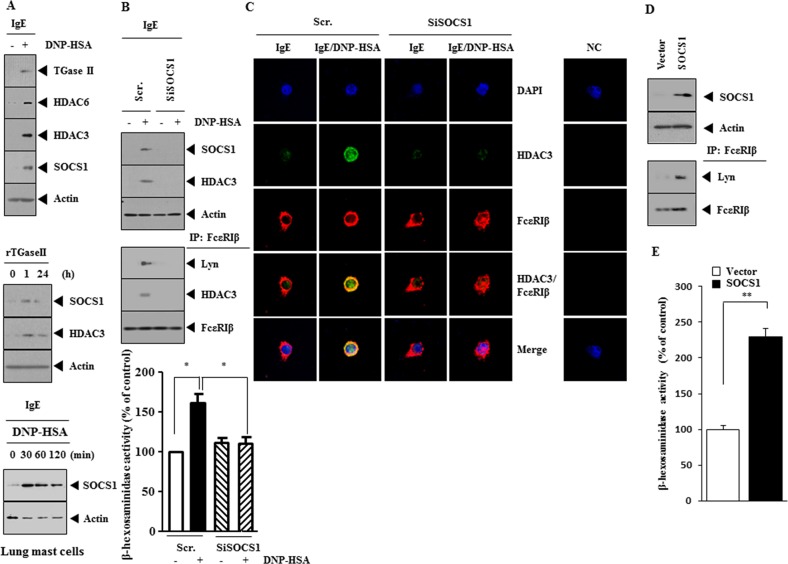
SOCS1 mediates allergic inflammation **(A)** The IgE (DNP-specific)-sensitized RBL2H3 cells were treated with DNP-HSA (100 ng/ml) for 1 h, followed by western blot (upper panel). RBL2H3 cells were treated with recombinant TGaseII protein (1 ng/ml), followed by western blot (middle panel). The IgE-sensitized lung mast cells were treated with DNP-HSA for various time intervals, followed by western blot (lower panel). **(B)** RBL2H3 cells were transfected with scrambled (Scr.) siRNA (10 nM) or SOCS1 siRNA (10 nM). The next day, cells were sensitized with IgE (100 ng/ml) for 24 h, followed by stimulation with DNP-HSA for 1 h. **(C)** Same as B except that immunofluorescence staining employing the indicated antibody was performed. **(D)** RBL2H3 cells were transfected with control vector (1 μg) or SOCS1 vector (1 μg). At 48 h after transfection, immunoprecipitation and western blot were performed. **(E)** Same as (D) except that β-hexosaminidase activity assays were performed. **, p<0.005.

Many reports suggest the role of SOCS1 in allergic inflammation. Antigen (DNP-HSA) stimulation increased the expression of SOCS1 along with hallmarks of allergic inflammation such as HDAC6, HDAC3 and TGase II in RBL2H3 cells (Figure 1A). HDAC activity regulates the expression of SOCS1 [19]. HDAC3 mediates allergic inflammation through its effect on the expression of MCP1 [20]. Recombinant TGaseII protein increased the expression of SOCS1 and HDAC3 (Figure 1A) and is known to induce features of allergic inflammation [21]. The downregulation of HDAC6 inhibited hall marks of allergic inflammation, such as increased expression of HDAC3, SOCS1, and Δ-hexosaminidase activity, in antigen-stimulated RBL2H3 cells (data not shown). Antigen-stimulated lung mast cells showed increased expression of SOCS1 (Figure 1A, lower panel). The down-regulation of SOCS1 prevented antigen from increasing the expression of HDAC3 (Figure 1B) and prevented antigen from inducing an interaction between FcεRIβ and Lyn and an interaction between FcεRIβ and HDAC3 (Figure 1B). The down-regulation of SOCS1 prevented antigen from increasing β-hexosaminidase activity in RBL2H3 cells (Figure 1B) and inhibited co-localization of FcεRIβ with HDAC3 (Figure 1C). SOCS1 induced an interaction between FcεRIβ and HDAC3 in an antigen-independent manner (Figure 1D) and increased β-hexosaminidase activity in an antigen-independent manner (Figure 1E). These results suggest the role of SOCS1 in allergic inflammation.

### SOCS1 mediates passive systemic anaphylaxis (PSA)

**Figure 2:**
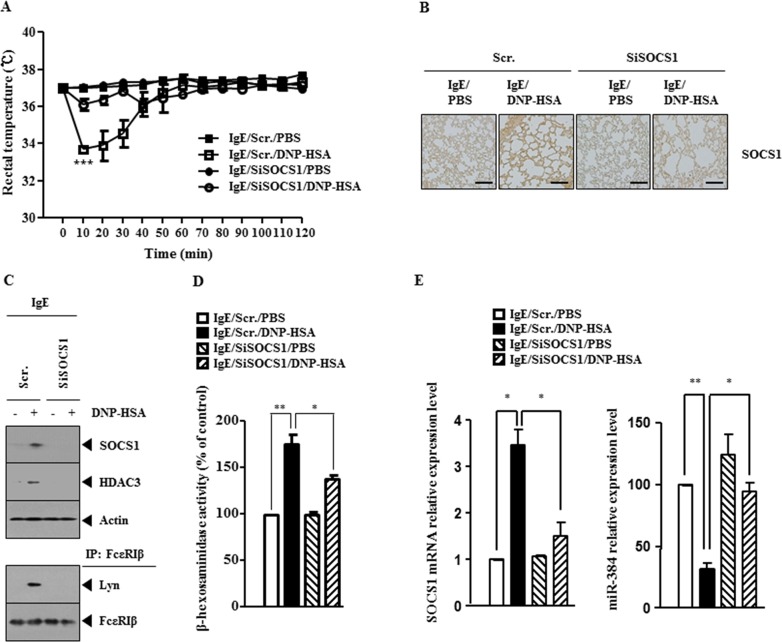
SOCS1 mediates passive systemic anaphylaxis (PSA) **(A)** BALB/c mice were injected intravenously with scrambled siRNA (100 nM) or SOCS1 siRNA (100 nM). The next day, BALB/c mice were injected intravenously with IgE (0.5 μg/kg). The following day, BALB/c mice were injected intravenously with DNP-HSA (250 μg/kg), and rectal temperatures were measured. Each experimental group consisted of five mice. The means ± S.E. of three independent experiments are depicted. **(B)** Immunohistochemical staining employing lung tissue was performed as described. **(C)** Tissue lysates were subjected to immunoprecipitation and western blot analysis. **(D)** Same as (C) except that β-hexosaminidase activity assays were performed. *, p<0.05; **, p<0.005. **(E)** Same as (D) except that qRT-PCR analysis was performed. *, p<0.05; **, p<0.005.

The role of SOCS1 in allergic inflammation was investigated. PSA decreased rectal temperatures of BALB/C mice (Figure 2A) and down-regulation of SOCS1 prevented antigen from decreasing rectal temperatures (Figure 2A). Immunohistochemical staining of lung tissue showed that antigen increased the expression of SOCS1 (Figure 2B). Western blot and immunoprecipitation employing lung tissue lysates indicate that down-regulation of SOCS1 prevented antigen from increasing the expression of HDAC3 and prevented antigen from inducing an interaction between FcεRIβ and Lyn (Figure 2C). Down-regulation of SOCS1 also prevented antigen from increasing β-hexosaminidase activity in lung tissue (Figure 2D). qRT-PCR analysis employing lung tissue lysates showed that down-regulation of SOCS1 prevented antigen from decreasing the expression of miR-384, a negative regulator of HDAC3 (Figure 2E). These results suggest the role of SOCS1 in anaphylaxis.

### SOCS1 mediates passive cutaneous anaphylaxis (PCA)

**Figure 3:**
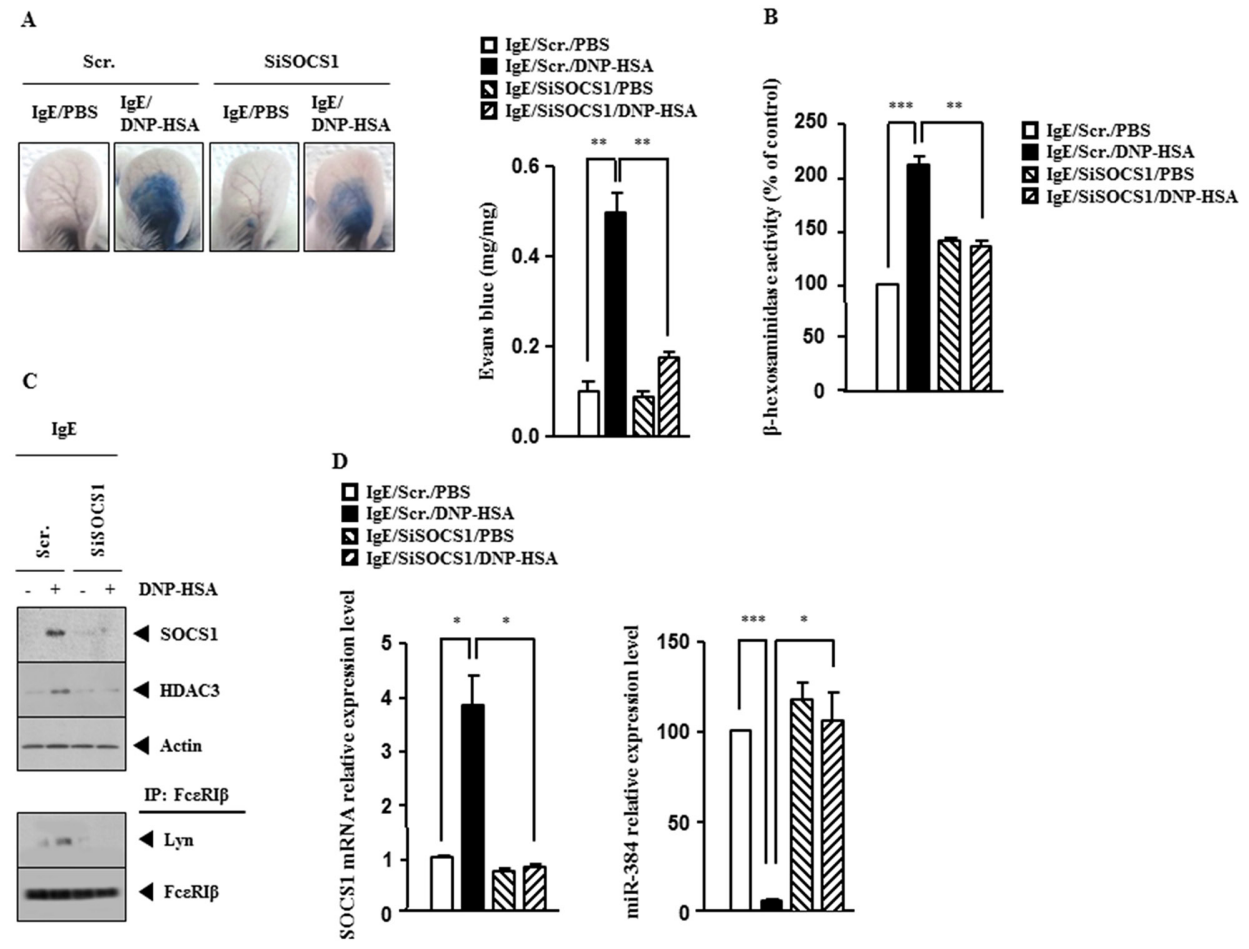
SOCS1 mediates passive cutaneous anaphylaxis (PCA) **(A)** BALB/c mice were given an intradermal injection of IgE (0.5 μg/kg) and intravenous injection of scrambled (100 nM) or SOCS1 siRNA (100 nM). The next day, BALB/c mice were given an intravenous injection of PBS or DNP-HSA (250 μg/kg) along with 2% (v/v) Evans blue solution. One hour after injection of Evans blue solution, the dye was eluted from the ear in 700 μl of formamide at 63°C. The absorbance was measured at 620 nm. Representative images from four animals of each experimental group are shown. **, p<0.005. **(B)** Ear tissue lysates from BALB/c mouse of each experimental group were subjected to β-hexosaminidase activity assay. **, p<0.005; ***, p<0.0005. **(C)** Same as (B) except that western blot and immunoprecipitation were performed. **(D)** Same as (B) except that qRT-PCR analysis was performed. *, p<0.05; ***, p<0.0005.

The BALB/C mouse model of passive cutaneous anaphylaxis (PCA) was employed to investigate the role of SOCS1 in anaphylaxis. Down-regulation of SOCS1 prevented antigen from increasing vascular permeability (Figure 3A) and prevented antigen from increasing β-hexosaminidase activity (Figure 3B). Western blot and immunoprecipitation employing ear tissue lysates showed that down-regulation of SOCS1 prevented antigen from increasing the expression of HDAC3 and prevented antigen from inducing an interaction between FcεRIβ and Lyn (Figure 3C). qRT-PCR analysis employing ear tissue lysates indicated that down-regulation of SOCS1 prevented antigen from decreasing the expression of miR-384 (Figure 3D). These results provide strong evidence that SOCS1 mediates *in vivo* allergic inflammation.

### miR-384-HDAC3 feedback loop regulates the expression of SOCS1 and allergic inflammation

miR-384 acts as a negative regulator of HDAC3 to inhibit allergic inflammation [22]. This led us to hypothesize that miR-384-HDAC3 feedback loop would regulate the expression of SOCS1. miR-384 inhibitor increased the expression of SOCS1 and HDAC3 and induced an interaction between FcεRIβ and Lyn in both an antigen-independent manner in RBL2H3 cells (Supplementary Figure 1A). miR-384 inhibitor increased the expression of HDAC3, SOCS1 and induced an interaction between FcεRIβ and Lyn in a SOCS1-dependent manner in RBL2H3 cells (Supplementary Figure 1B). miR-384 inhibitor increased β-hexosaminidase activity in a SOCS1-dependent manner in RBL2 H3 cells (Supplementary Figure 1C), and increased vascular permeability and β-hexosaminidase activity in a SOCS1-dependent manner in BALB/C mice (Supplementary Figure 1D). miR-384 inhibitor also increased the expression of SOCS1 and induced an interaction between FcεRIβ and Lyn in BALB/C mice (Supplementary Figure 1E). Antigen induced the binding of HDAC3 and YY1 to the promoter sequences of SOCS1 in RBL2H3 cells (Supplementary Figure 1F) while miR-384 mimic, a negative regulator of HDAC3, prevented the binding of HDAC3 and YY1 to the promoter sequences of SOCS1 (Supplementary Figure 1G). Promoter analysis reveals potential binding sites for various other transcriptional factors, such as SP1, SNAIL, YY1, in the promoter sequences of SOCS1 (personal observations). Antigen induced an interaction between HDAC3 and SOCS1 and an interaction between FcεRIβ and SOCS1 in RBL2H3 cells (Supplementary Figure 1H), suggesting that HDAC3 and SOCS1 form complex to bind to the promoter sequences of SOCS1. These results suggest that miR-384-HDAC3 feedback loop regulates the expression of SOCS1.

### miR-122-SOCS1 negative feedback loop regulates the allergic inflammation

**Figure 4:**
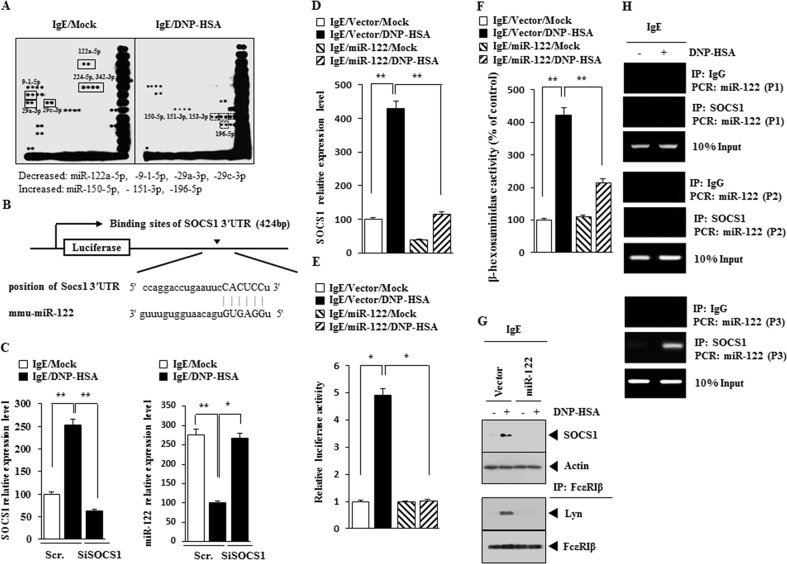
miR-122-SOC1 negative feedback loop regulates the allergic inflammation **(A)** The IgE-sensitized RBL2H3 cells were treated with DNP-HSA for 1 h, followed by miRNA array analysis. **(B)** Shows the potential binding site of miR-122 to 3′UTR-SOCS1. **(C)** RBL2H3 cells were transfected with scrambled siRNA or SOCS1 siRNA. The next day, cells were sensitized with IgE for 24 h, followed by stimulation with DNP-HSA. One hour after stimulation with DNP-HSA, cell lysates were subjected to qRT-PCR analysis. *, p<0.05; **, p<0.005. **(D)** Same as (C) except that RBL2H3 cells were transfected with control vector or miR-122 vector. **, p<0.005. **(E)** Same as (D) except that luciferase activity assays were performed. *, p<0.05. **(F)** Same as (E) except that β-hexosaminidase activity assays were performed. **, p<0.005. **(G)** Same as (F) except that western blot and immunoprecipitation were performed. **(H)** The IgE-sensitized RBL2H3 cells were treated with DNP-HSA for 1h, followed by ChIP assays. P1, P2 and P3 denote region of miR-122 promoter amplified by primer 1, 2 and 3, respectively

The silencing of Dicer, a key enzyme of miRNA biogenesis, attenuates degranulation, indicating that miRNAs are involved in mast cell degranulation [23]. miRNAs, such as miR-122, -224-5p and 342-3p, were decreased by antigen in RBL2H3 cells (Figure 4A). TargetScan analysis predicted the binding of miR-122 to the 3′-UTR of SOCS1 (Figure 4B). qRT-PCR analysis showed that the down-regulation of SOCS1 increased the expression of miR-122 in antigen-stimulated RBL2H3 cells (Figure 4C). miR-122 prevented antigen from increasing the expression of SOCS1 mRNA (Figure 4D) and negatively regulated luciferase activity associated with wild type 3′-UTR of SOCS1 (Figure 4E). miR-122 prevented antigen from increasing β-hexosaminidase activity in RBL2H3 cells (Figure 4F) and also prevented antigen from increasing the expression of SOCS1 as wells preventing antigen from inducing an interaction between FcεRIβ and Lyn in RBL2H3 cells (Figure 4G). Antigen induced the binding of SOCS1 to the promoter sequences of miR-122 (Figure 4H). Promoter analysis reveals potential binding sites for various other transcriptional factors, such as STAT, C-JUN, HDAC3, in the promoter sequences of miR-122 (personal observations). These results suggest that miR-122-SOCS1 feedback loop regulates allergic inflammation.

### miR-122 inhibitor targets SOCS1 to induce features of allergic inflammation

miR-122 inhibitor increased the expression of SOCS1 and induced an interaction between FcεRIβ and Lyn in SOCS1-dependent manner in RBL2H3 cells (Supplementary Figure 2A). miR-122 inhibitor increased the expression of SOCS1 at the transcriptional level (Supplementary Figure 2B) and β-hexosaminidase activity in SOCS1-dependent manner (Supplementary Figure 2C). miR-122 inhibitor increased vascular permeability (Supplementary Figure 2D) and increased β-hexosaminidase activity in an SOCS1-dependent manner in BALB/C mice (Supplementary Figure 2E). miR-122 inhibitor increased the expression of SOCS1 and induced an interaction between FcεRIβ and Lyn in BALB/C mice (Supplementary Figure 2F). These results suggest that miR-122 inhibitor induces the *in vivo* features of allergic inflammation in a SOCS1-dependent manner.

### SOCS1 mediates the interaction between cancer cells and mast cells

**Figure 5:**
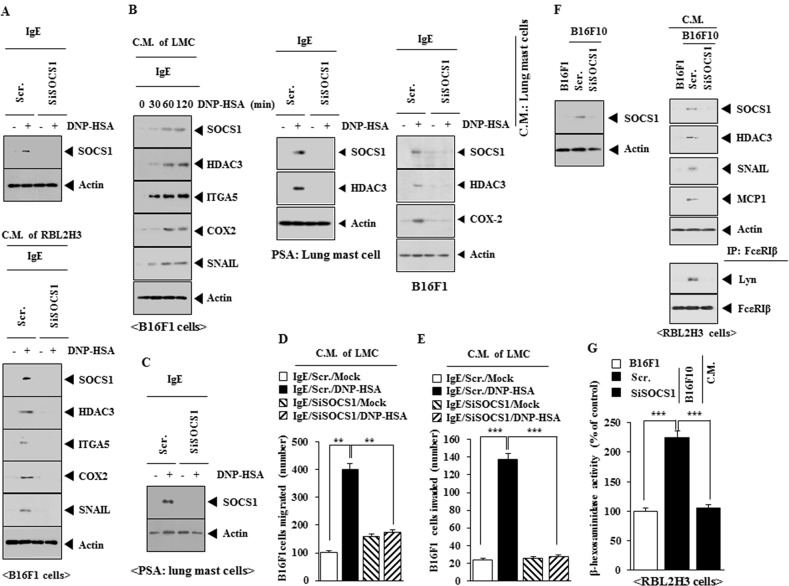
SOCS1 mediates the interaction between cancer cells and mast cells **(A)** RBL2H3 cells were transfected with scrambled siRNA or SOCS1 siRNA. The next day, cells were sensitized with IgE for 24 h, followed by stimulation with DNP-HSA for 1 h. cell lysates were subjected to western blot (upper panel). RBL2H3 cells were transfected with scrambled siRNA or SOCS1 siRNA. The next day, cells were sensitized with IgE for 24 h, followed by stimulation with DNP-HSA. One hour after stimulation with DNP-HSA, conditioned medium was obtained and added to B16F1 cells for 8 h, followed by western blot (lower panel). **(B)** The IgE-sensitized lung mast cells were stimulated with DNP-HSA for various time intervals. The conditioned medium obtained at each time point was added to B16F1 cells for 8 h, followed by western blot analysis. Animal model of PSA was performed in the absence or presence of SOCS1 siRNA (100 nM). Western blot analysis was performed employing lung mast cells. The conditioned medium of lung mast cells was obtained and added to B16F1 cells for 8 h, followed by western blot. **(C)** BALB/c mice were injected intravenously with scrambled siRNA (100 nM) or SOCS1 siRNA (100 nM). The next day, BALB/c mice were injected intravenously with IgE (0.5 μg/kg). The following day, BALB/c mice were injected intravenously with DNP-HSA (250 μg/kg). Lung mast cell lysates were subjected to western blot analysis. **(D)** The conditioned medium of lung mast cells obtained after PSA was added to B16F1 cells and wound migration assays were performed. **, p<0.005. **(E)** Same as (D) except that chemoinvasion assays were performed. ***, p<0.0005. **(F)** B16F10 cells were transfected with the indicated siRNA. At 48 h after transfection, western blot was performed (upper panel). The indicated cancer cells were transfected with the indicated siRNA. The conditioned medium was added to RBL2H3 cells for 8 h, followed by immunoprecipitation and western blot analysis. **(G)** Same as (F) except that β-hexosaminidase activity assays were performed. ***, p<0.0005.

Allergic inflammation enhances the tumorigenic and the metastatic potential of cancer cell via cellular interactions [21]. Down-regulation of SOCS1 decreased the expression of SOCS1 in RBL2H3 cells (Figure 5A). The conditioned medium of antigen-stimulated RBL2H3 cells, when added to B16F1 cells, increased the expression of HDAC3, integrin α5, COX- 2 and SNAIL in a SOCS1-dependent manner in B16F1 cells (Figure 5A). Similarly, the conditioned medium of antigen-stimulated lung mast cells, when added to B16F1 cells, increased the expression of HDAC3, integrin α5, COX- 2 and SNAIL in a SOCS1-dependent manner in B16F1 cells (Figure 5B). PSA increases the expression of SOCS1 and HDAC3 in lung mast cells in a SOCS1-dependent manner (Figure 5B). The conditioned medium of lung mast cells obtained after the induction of PSA increases the expression of SOCS1, HDAC3 and COX-2 in B16F1 cells in a SOCS1-dependent manner (Figure 5B). PSA increased the expression of SOCS1 in lung mast cells (Figure 5C). The conditioned medium of lung mast cells obtained after PSA induction, when added to B16F1 cells, increased the migration potential (Figure 5D) and invasion potential (Figure 5E) of B16F1 cells, in a SOCS1- dependent manner. The conditioned medium of highly malignant B16F10 cells, but not conditioned medium of B16F1 cells, when added to RBL2H3 cells, increased the expression of SOCS1, HDAC3, SNAIL and MCP1, and induced an interaction between FcεRIβ and Lyn in a SOCS1-dependent manner (Figure 5F). The conditioned medium of B16F10 cells, but not conditioned medium of B16F1 cells, when added to RBL2H3 cells, increased β-hexosaminidase activity in a SOCS1-depedndent manner (Figure 5G). These results suggest that SOCS1 regulates the interaction between cancer cells and mast cells.

### miR-122 inhibits the cellular interaction

Expression of SOCS1 was higher in B16F10 cells than in B16F1 cells (Supplementary Figure 3A) and expression of SOCS1 in B16F10 cells was decreased by miR-122 (Supplementary Figure 3A). miR-122 also prevented the conditioned medium of B16F10 cells from increasing the expression of hall marks of allergic inflammation, such as HDAC3, SOCS1 and SNAIL, and inducing an interaction between FcεRIβ and Lyn in RBL2H3 cells (Supplementary Figure 3B). miR-122 prevented the conditioned medium of B16F10 cells from increasing β-hexosaminidase activity in RBL2H3 cells (Supplementary Figure 3C). miR-122 prevented antigen from increasing the expression of SOCS1 and MCP1 in RBL2H3 cells (Supplementary Figure 3D). miR-122 prevented the conditioned medium of antigen-stimulated RBL2H3 cells from increasing the expression of HDAC3, SOCS1, TGaseII and COX-2 in B16F1 cells (Supplementary Figure 3D). miR-122 also prevented the conditioned medium of antigen-stimulated RBL2H3 cells from enhancing the invasion and migration potential of B16F1 cells (Supplementary Figure 3E). miR-122 also prevented the conditioned medium of antigen-stimulated RBL2H3 cells from increasing the expression of SOCS1 mRNA in B16F1 cells (Supplementary Figure 3F). These results suggest that miR-122 inhibits the cellular interaction.

### SOCS1 is necessary for the enhanced tumorigenic potential of B16F1 cells by allergic inflammation

PSA enhanced the tumorigenic potential of B16F1 cells in SOCS1-dependent manner (Supplementary Figure 4A). PSA increased expression of HDAC3, COX2, TGaseII and MCP1 in a SOCS1-dependent manner in lung tumor tissue (Supplementary Figure 4B). PSA also induced an interaction between FcεRIβ and Lyn in SOCS1-dependent manner in lung tumor tissue (Supplementary Figure 4B). The down-regulation of SOCS1 increased the expression of miR-384 and miR-122 at the transcriptional level (Supplementary Figure 4C) and decreased β-hexosaminidase activity in lung tumor tissue (Supplementary Figure 4D). These results suggest that SOCS1 mediates allergic inflammation-promoted enhanced tumorigenic potential of cancer cells through its effect on the expression of miR-122.

### SOCS1 is necessary for the enhanced metastatic potential of cancer cells by allergic inflammation

**Figure 6:**
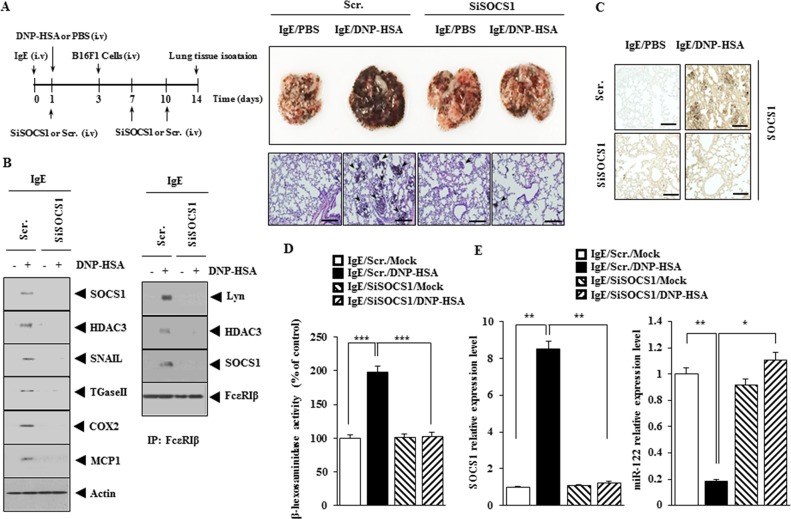
SOCS1 is necessary for the enhanced metastatic potential of cancer cells by allergic inflammation **(A)** BALB/C mice were sensitized to IgE (0.5 μg/kg) by an intravenous injection. The next day, BALB/C mice were given an intravenous injection of DNP-HSA (250 μg/kg). Each mouse received an injection of B16F1 melanoma cells (2 × 10^5^) on the day 3 of the time line. BALB/C mice were given an intravenous injection with the indicated siRNA (100 nM) on days 1, 7, and 10 of the time line. On day 14 of the time line, lung tissues were harvested. Formalin-fixed lung sections were stained with H&E. Black arrows denote lung metastatic foci (scale bar, 100 μm). The extent of lung metastasis was determined as described. **(B)** Lung tumor tissue lysates from each mouse of each experimental group of mice were subjected to western blot and immunoprecipitation. **(C)** Immunohistochemical staining of lung tumor tissue was performed (scale bar, 100 μm). **(D)** Lung tumor tissue lysates were subjected to β-hexosaminidase activity assays. ***, <0.0005. **(E)** Same as (D) except that qRT-PCR analysis was performed. *, p<0.05; **, p<0.005.

Next, the effect of SOCS1 on the metastatic potential of cancer cells was examined. PSA enhanced the metastatic potential of B16F1 cells in SOCS1-dependent manner (Figure 6A). The down-regulation of SOCS1 decreased the expression of HDAC3, TGaseII, MCP1, SNAIL, COX-2 and the interaction between FcεRIβ and Lyn in lung tumor tissue (Figure 6B). Immunohistochemical staining of lung tumor tissue showed that PSA increased the expression of SOCS1 (Figure 6C). Down-regulation of SOCS1 decreased β-hexosaminidase activity (Figure 6D) while increasing the expression of miR122 in lung tumor tissue (Figure 6E). These results suggest that SOCS1 mediates the enhanced metastatic potential of cancer cells by allergic inflammation through its effect on the expression of miR-122.

### SOCS1 is necessary for the tumorigenic potential of B16F10 melanoma cells and the cellular interaction

Because SOCS1 expression was higher in B16F10 cells than in B16F1 cells (Supplementary Figure 3A), we next examined whether SOCS1 would regulate the tumorigenic potential of B16F10 cells. Down-regulation of SOCS1 decreased the tumorigenic potential of B16F10 cells (Supplementary Figure 5A). Down-regulation of SOCS1 decreased β-hexosaminidase activity while increasing the expression of miR-122 in tumor tissue (Supplementary Figure 5B). Down-regulation of SOCS1 decreased the expression of HDAC3, TGaseII and COX-2 and inhibited the interactions of FcεRIβ with Lyn, HDAC3 and SOCS1 in tumor tissue (Supplementary Figure 5C). These results indicate that SOCS1 is necessary for the tumorigenic potential of B16F10 melanoma cells and the cellular interaction.

### SOCS1 mimetic peptide inhibits *in vitro* allergic inflammation

**Figure 7:**
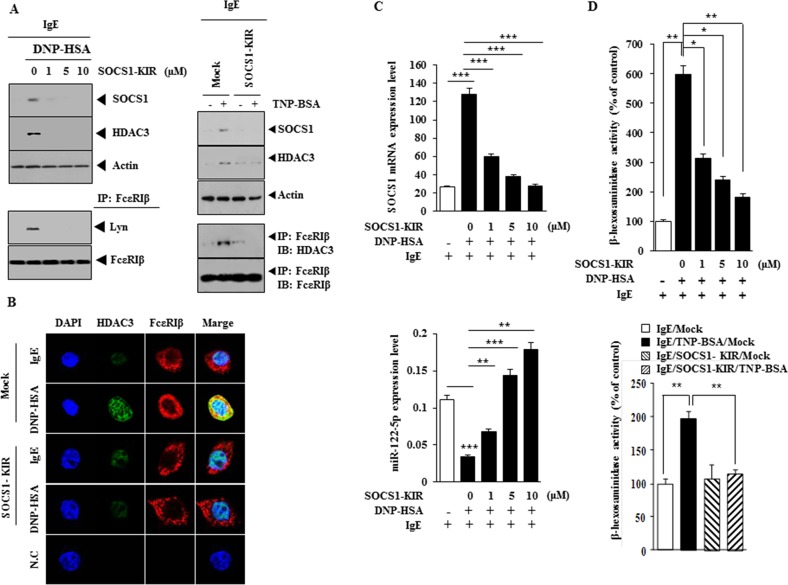
SOCS1-KIR mimetic peptide inhibits features of *in vitro* allergic inflammation **(A)** The IgE-sensitized RBL2H3 cells were pretreated with various concentrations of SOCS1-KIR for 1 h, followed by stimulation with DNP-HSA (left panel). The IgE (TNP-specific)-sensitized RBL2H3 cells were pretreated with SOCS1-KIR (1 μM) for 1 h, followed by stimulation with TNP-BSA (100 ng/ml) for 1 h (right panel). Western blot and immunoprecipitation were performed. **(B)** The IgE-sensitized RBL2H3 cells were pretreated with SOCS1-KIR for 1 h, followed by immunofluorescence staining. **(C)** Same as (A) except that qRT-PCR analyses were performed. **, p<0.005; ***, p<0.0005. **(D)** Same as (A) except that β-hexosaminidase activity assays were performed. *, p<0.05; **, p<0.005.

SOCS1 mimetic peptide (SOCS1-KIR; SOCS1 Kinase Inhibitory Region) inhibits ocular inflammation [17]. We therefore examined the effect of SOCS1-KIR on the allergic inflammation. SOCS1-KIR decreased the expression of SOCS1 and HDAC3 and inhibited interactions of FcεRIβ with HDAC3 and Lyn in RBL2H3 cells (Figure 7A). SOCS1-KIR decreased the expression of HDAC3 and inhibited a co-localization of FcεRIβ with HDAC3 (Figure 7B). SOCS1-KIR decreased SOCS1 mRNA expression level (Figure 7C) and restored the expression of miR-122 in a dose-dependent manner (Figure 7C). SOCS1-KIR decreased the β-hexosaminidase activity in antigen-stimulated RBL2H3 cells (Figure 7D). These results provide strong evidence for the role of SOCS1 in allergic inflammation.

### SOCS1 mimetic peptide inhibits PCA

**Figure 8:**
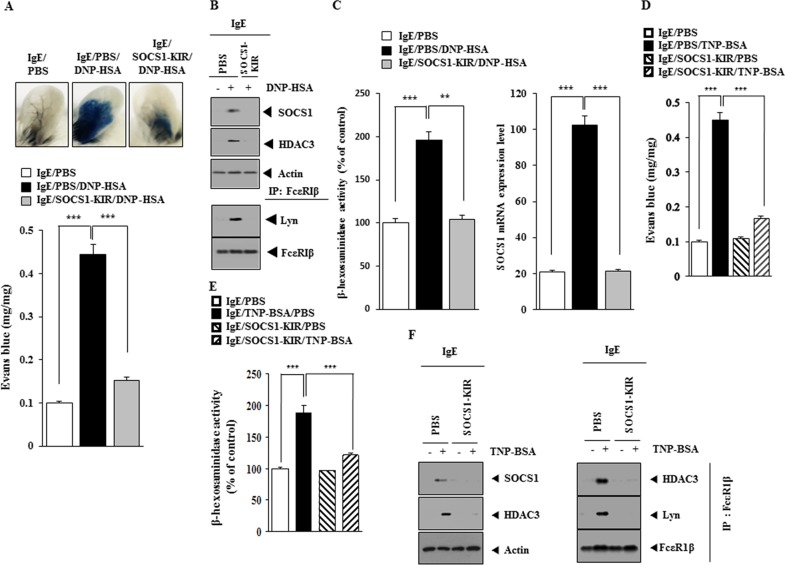
SOCS1-KIR mimetic peptide inhibits PCA **(A)** BALB/C mice were given an intradermal injection of IgE antibody (0.5 μg/kg) or IgG (0.5 μg/kg). The next day, BALB/C mice were given an intravenous injection of PBS or DNP-HSA (250 μg/kg) along with SOCS1-KIR (9 mg/kg) along with 2% (v/v) Evans blue solution. One hour after the injection, the extent of vascular permeability was determined as described. Means ± S.E. of three independent experiments are depicted. ***, p<0.0005. Each experimental group consisted of four mice. **(B)** Ear tissue lysates from each mouse of each experimental group of mice were subjected to western blot and immunoprecipitation. **(C)** qRT-PCR and β-hexosaminidase activity assays were performed. **, p<0.005; ***, p<0.0005. **(D)** BALB/C mice were given an intradermal injection of TNP-specific IgE (0.5 μg/kg) or TNP-specific IgG (0.5 μg/kg). The next day, BALB/C mice were given an intravenous injection of PBS or TNP-BSA (250 μg/kg) along with SOCS1-KIR (9 mg/kg). The extent of vascular permeability was determined as described. ***, p<0.0005. Each experimental group consisted of four mice. **(E)** Ear tissue lysates from each mouse of each experimental group of mice were subjected to β-hexosaminidase activity assays. ***, p<0.0005. **(F)** Ear tissue lysates were subjected to western blot and immunoprecipitation.

Next, the effect of SOCS1-KIR on allergic inflammation was examined. SOCS1-KIR exerted a negative effect on the increased vascular permeability in a mouse model of PCA (Figure 8A). SOCS1-KIR prevented antigen from increasing the expression of SOCS1 and HDAC3 and prevented antigen from inducing an interaction between FcεRIΔ and Lyn in a mouse model of PCA (Figure 8B). SOCS1-KIR prevented antigen from increasing β-hexosaminidase activity and prevented antigen from increasing the expression of SOCS1 mRNA in mouse model of PCA (Figure 8C). SOCS1-KIR prevented antigen (TNP-BSA) from increasing vascular permeability (Figure 8D) and prevented antigen from increasing β-hexosaminidase activity in mouse model of PCA employing TNP-specific IgE (Figure 8E). SOCS1-KIR also prevented antigen from increasing the expression of SOCS1, HDAC3 and prevented antigen from inducing an interaction between FcεRIΔ and Lyn in a mouse model of PCA employing TNP-specific IgE (Figure 8F).

### SOCS1-KIR mimetic peptide inhibits PSA

The effect of SOCS1-KIR on PSA was examined. SOCS1-KIR prevented antigen from decreasing rectal temperature in mouse model of PSA (Supplementary Figure 6A). SOCS1-KIR prevented antigen from increasing the expression of HDAC3, TGaseII and SOCS1 and prevented antigen from inducing an interaction of FcεRIβ with TGaseII, HDAC3 and Lyn in mouse model of PSA (Supplementary Figure 6B). SOCS1-KIR also prevented antigen from increasing β-hexosaminidase activity in a mouse model of PSA (Supplementary Figure 6C).

### SOCS1-KIR mimetic peptide inhibits allergic inflammation-promoted enhanced tumorigenic potential of B16F1 melanoma cells

SOCS1-KIR exerted a negative effect on the enhanced tumorigenic potential of B16F1 melanoma cells by PSA (Supplementary Figure 7A). SOCS1peptide prevented antigen from increasing β-hexosaminidase activity in tumor tissue (Supplementary Figure 7B) and prevented antigen from increasing the expression of SOCS1 and HDAC3 in tumor tissue (Supplementary Figure 7C). SOCS1-KIR also prevented antigen from inducing an interaction between FcεRIβ and HDAC3 (Supplementary Figure 7C).

### SOCS1-KIR mimetic peptide inhibits allergic inflammation-promoted enhanced metastatic potential of B16F1 melanoma cells

**Figure 9:**
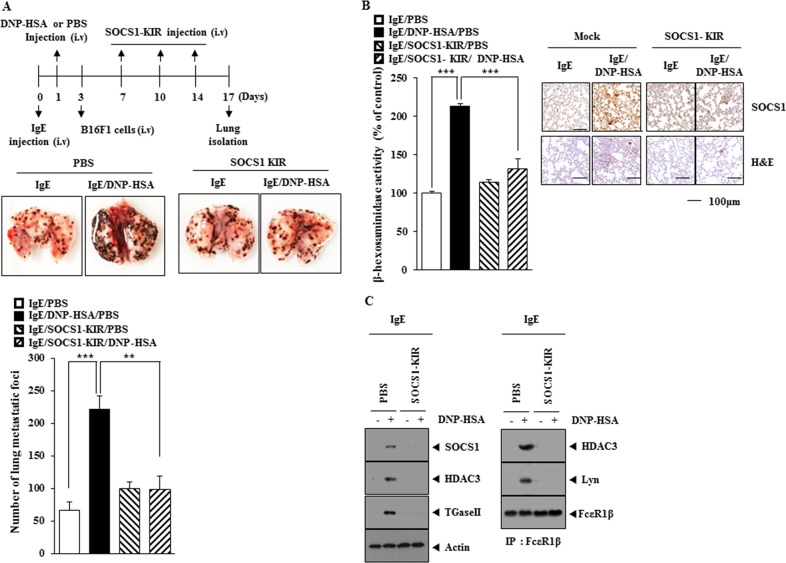
SOCS1-KIR mimetic peptide inhibits allergic inflammation-promoted enhanced metastatic potential of B16F1 melanoma cells **(A)** BALB/C mice were sensitized with IgE (0.5 μg/kg) by an intravenous injection. The next day, BALB/C mice were given an intravenous injection of DNP-HSA (250 μg/kg). Each mouse received intravenous injection of B16F1 melanoma cells (2×10^5^) on the day 3 of the time line and received intravenous injection of SOCS1-KIR (9 mg/kg) at the indicated day of the time line. On the day 17 of the time line, tumor tissues were harvested. **, p<0.005; ***, p<0.0005. **(B)** Tumor tissue lysates were subjected to β-hexosaminidase activity assays. ***, p<0.0005. **(C)** Lung tumor tissue lysates were subjected to western blot and immunoprecipitation.

SOCS1-KIR exerted a negative effect on the enhanced the metastatic potential of B16F1 melanoma cells by PSA (Figure 9A). SOCS1peptide prevented antigen from increasing β-hexosaminidase activity in tumor tissue (Figure 9B). Immunohistochemical staining of lung tumor tissue showed that SOCS1-KIR prevented antigen from increasing the expression of SOCS1 (Figure 9B). SOCS1-KIR also prevented antigen from increasing the expression of SOCS1, HDAC3 and TGaseII and also prevented antigen from inducing an interaction of FcεRIβ with HDAC3 and Lyn in tumor tissue (Figure 9C).

### SOCS1-KIR inhibits cellular interactions between cancer cells and mast cells

**Figure 10:**
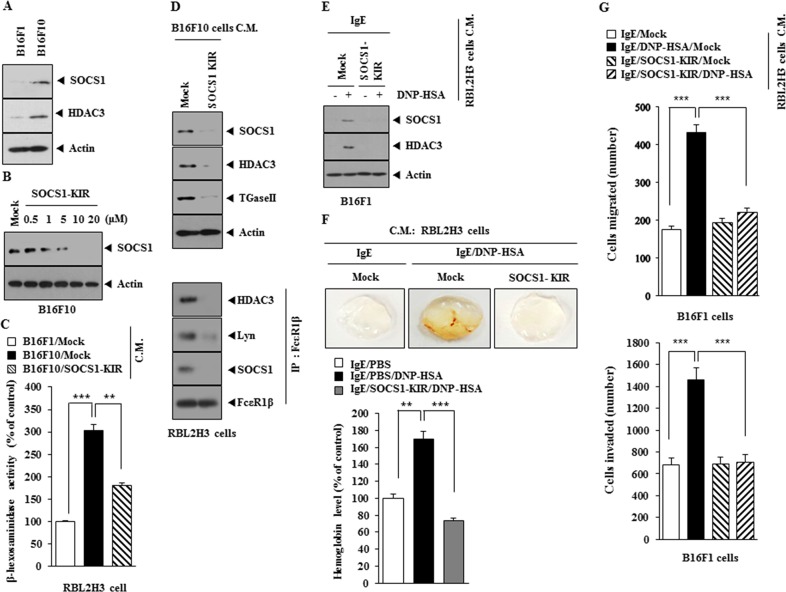
SOCS1-KIR inhibits cellular interaction during allergic inflammation-promoted tumorigenesis **(A)** Western blot was performed. **(B)** B16F10 cells were treated with various concentrations of SOCS1-KIR for 24 h, followed by western blot. **(C)** The conditioned medium of B16F10 cells, treated with SOCS1-KIR for 24 h, was added to RBL2H3 cells for 24 h, followed by β-hexosaminidase activity assays. **, p<0.005; ***, p<0.0005. **(D)** The conditioned medium of B16F10 cells, treated with SOCS1-KIR for 24 h, was added to RBL2H3 cells for 24 h, followed by western blot and immunoprecipitation. **(E)** The conditioned medium of antigen-stimulated RBL2H3 cells treated with or without SOCS1-KIR (5 μM) for 2 h was added to B16F1 cells for 24 h, followed by western blot analysis. **(F)** The conditioned medium of antigen-stimulated RBL2H3 cells treated with or without SOCS1-KIR (5 μM) for 2 h was subjected to matrigel plug assays. **, p<0.005; ***, p<0.0005. **(G)** The indicated conditioned medium of RBL2H3 cells was added to B16F1 cells. Invasion and migration potential of B16F1 cells were determined. ***, p<0.0005.

Expression levels of SOCS1 and HDAC3 were higher in B16F10 cells than in B16F1 cells (Figure 10A). SOCS1-KIR decreased the expression of SOCS1 in a dose-dependent manner in B16F10 cells (Figure 10B). When added to RBL2H3 cells, the conditioned medium of B16F10 cells obtained in the absence of treatment with SOCS1-KIR, increased β-hexoasmianidase activity (Figure 10C) as well as the expression of SOCS1, HDAC3 and TGase II, and induced interactions of FcεRIβ with HDAC3, Lyn and SOCS1 (Figure 10D). The conditioned medium of antigen-stimulated RBL2H3 cells, obtained in the absence of treatment with SOCS1-KIR, increased the expression of SOCS1 and HDAC3 in B16F1 cells (Figure 10E). The matigel plug assay showed the angiogenic potential of antigen-stimulated RBL2H3 cells in the absence of SOCS1-KIR (Figure 10F). The conditioned medium of antigen-stimulated RBL2H3 cells obtained in the absence of treatment with SOCS1-KIR, when added to B16F1 cells, enhanced the invasion and migration potential of B16F1 cells (Figure 10G). These results suggest that SOCS1 mediates the interaction between cancer cells and mast cells during allergic inflammation-promoted enhanced tumorigenic and metastatic potential.

### Mast cells and macrophages form positive feedback loop during allergic inflammation

**Figure 11:**
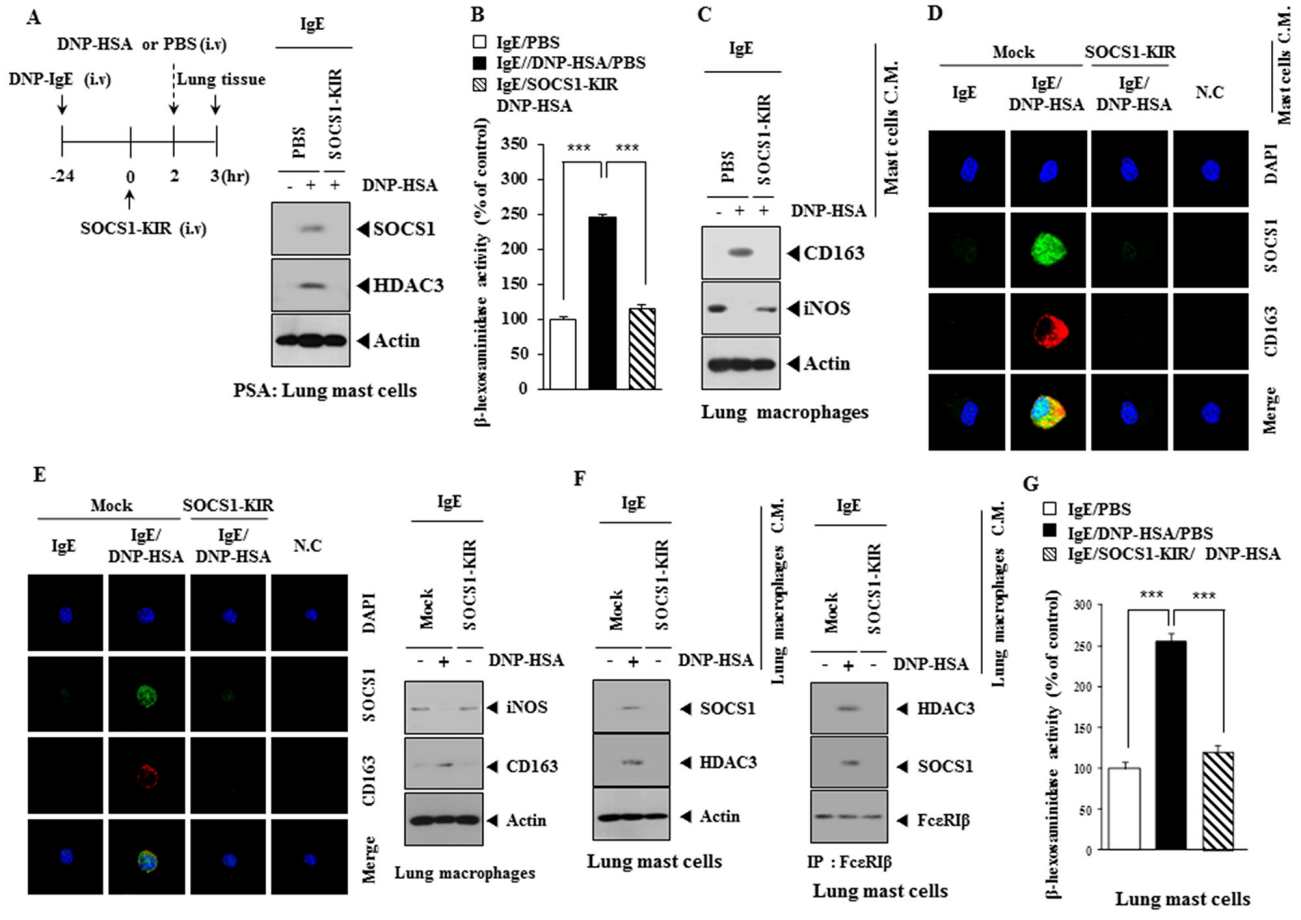
Mast cells and macrophages form positive feedback loop in allergic inflammation **(A)** PSA employing BALB/c mouse was performed in the absence or presence of SOCS1-KIR. Lung mast cell lysates were subjected to western blot analysis. **(B)** Same as (A) except that β-hexosaminidase activity assays were performed. ***, p<0.0005. **(C)** The conditioned medium of lung mast cells isolated after PSA in the absence or presence of SOCS1-KIR was added to mouse macrophages for 1 h, followed by western blot analysis. **(D)** Same as (C) except that immunofluorescence staining was performed. **(E)** Lung macrophages obtained after PSA, in the absence or presence of SOCS1-KIR, were subjected to immunofluorescence staining (left panel). Cell lysates were subjected to western blot (right panel). **(F)** The conditioned medium of lung macrophages obtained after PSA, was added to lung mast cells for 24 h, followed by western blot and immunoprecipitation. **(G)** Same as (F) except that β-hexosaminidase activity assays were performed. ***, p<0.0005.

Expression of HDAC3, SOCS1 (Figure 11A) and β-hexosaminidase activity (Figure 11B) was increased in lung mast cells after PSA induction, but not after PSA in the presence of SOCS1-KIR. The conditioned medium of lung mast cells obtained after PSA induction in the absence of SOCS1-KIR treatment increased the expression of CD163 while decreasing the expression of iNOS in lung macrophages (Figure 11C). When conditioned medium of lung mast cells obtained after PSA induction in the absence of SOCS1-KIR treatment was added to lung macrophages, expression of CD163 and SOCS1 was increased and induced a co-localization of SOCS1 with CD163 was induced (Figure 11D). Expression of iNOS was decreased in lung macrophages obtained after PSA induction in the absence of SOCS1-KIR treatment, while expression of CD163 was increased (Figure 11E). When conditioned medium of lung macrophages obtained after PSA induction in the absence of SOCS1-KIR treatment was added to lung mast cells, the expression of SOCS1 and HDAC3 increased, and interaction of FcεRIβ with HDAC3 and SOCS1 was induced (Figure 11F). When added to lung mast cells, the conditioned medium of lung macrophages obtained after PSA induction, increased β-hexosaminidase activity (Figure 11G). These results indicate that mast cells and macrophages form a positive feedback loop during allergic inflammation.

### JAK2 binds to SOCS1-KIR and mediates allergic inflammation

**Figure 12:**
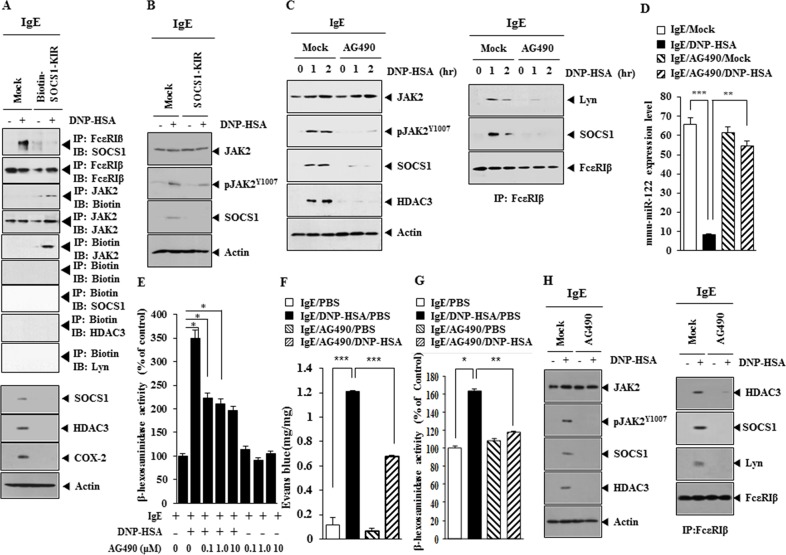
JAK2 binds to SOCS1-KIR and mediates allergic inflammation **(A)** The IgE-sensitized RBL2H3 cells were pretreated with SOCS1-KIR for 2 h, followed by stimulation with DNP-HSA. Cell lysates were subjected to immunoprecipitation employing the indicated antibodies and western blot. **(B)** The IgE-sensitized RBL2H3 cells were pretreated with SOCS1-KIR for 2 h, followed by stimulation with DNP-HSA. Western blot was performed. **(C)** The IgE-sensitized RBL2H3 cells were pretreated with JAK2 inhibitor AG490 (0.1 μM) for various time intervals, followed by stimulation with DNP-HSA for 1h, followed by western blot and Immunoprecipitation. **(D)** The IgE-sensitized RBL2H3 cells were pretreated with AG490 for 1 h, followed by stimulation with DNP-HSA for 1 h. qRT-PCR analysis was performed. **, p<0.005; ***, p<0.0005. **(E)** The IgE-sensitized RBL2H3 cells were pretreated with various concentration of AG490 for 1 h, followed by stimulation with DNP-HSA for 1 h. β-hexosaminidase activity assays were performed. *, p<0.05. **(F)** BALB/C mice were given an intradermal injection of IgE (0.5 μg/kg). The next day, BALB/C mice were given an intravenous injection of DNP-HSA (250 μg/kg) along with AG490 (0.15 mg/kg). The extent of vascular permeability was determined. Each experimental group consisted of four mice. ***, p<0.0005. **(G)** Ear tissue lysates were from each mouse of each experimental group of mice and were subjected to β-hexosaminidase activity assays. *, p<0.05; **, p<0.005. **(H)** Ear tissue lysates were subjected to western blot and immunoprecipitation.

SOCS1-KIR binds to JAK2 and exhibits anti-inflammatory activity [17], therefore the effect of JAK2 on allergic inflammation was investigated. SOCS1-KIR inhibited the interaction between FcεRIβ and SOCS1 in antigen-stimulated RBL2H3 cells (Figure 12A). Biotin-SOCS1-KIR bound to JAK2 (Figure 12A), but not to HDAC3, SOCS1 or Lyn (Figure 12A). Biotin-SOCS1-KIR decreased the expression of SOCS1, HDAC3 and COX-2 in antigen-stimulated RBL2H3 cells (Figure 12A). SOCS1-KIR prevented antigen from increasing the expression of pJAK2^Y1007^ (Figure 12B). An inhibitor of JAK2 (AG490) prevented antigen from increasing the expression of SOCS1, HDAC3 and pJAK2^Y1007^ and interactions of FcεRIβ with Lyn and SOCS1 in RBL2H3 cells (Figure 12C). AG490 also prevented antigen form increasing the expression of pSTAT1^Y701^ and pSTAT3^Y705^ (data not shown). AG490 restored the expression of miR-122 in RBL2H3 cells (Figure 12D) and prevented antigen from increasing β-hexosaminidase activity (Figure 12E). AG490 prevented antigen from increasing vascular permeability (Figure 12F) and prevented antigen from increasing β-hexosaminidase activity in a mouse model of PCA (Figure 12G). AG490 prevented antigen from increasing the expression of pJAK2^Y1007^, HDAC3 and SOCS1 and prevented antigen from inducing an interactions of FcεRIβ with HDAC3, SOCS1 and Lyn in a mouse model of PCA (Figure 12H). These results indicate that the miR-122-SOCS1-JAK2 axis regulates allergic inflammation.

### SOCS1 and JAK2 inhibits the production of anti-allergic TGF-β1

**Figure 13:**
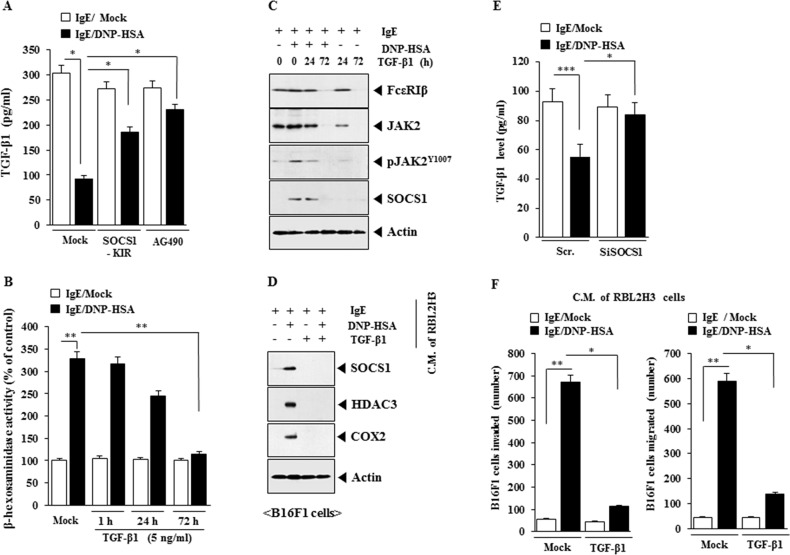
SOCS1 and JAK2 regulate the production of anti-allergic TGF-β1 **(A)** The IgE-sensitized RBL2H3 cells were pretreated with SOCS1-KIR (5 μM) or AG490 (0.1 μM) for 3 h, followed by stimulation with DNP-HSA. TGF-β1 level was measured. *, p<0.05. **(B)** RBL2H3 cells were treated with TGF-β1 (5 ng/ml) for various time intervals as indicated. Cells were then sensitized with IgE for 24 h, followed by stimulation with DNP-HSA for 1 h. β-hexosaminidase activity assays were performed. **, p<0.005. **(C)** RBL2H3 cells were treated with TGF-β1 (5 ng/ml) for various time intervals as indicated. Cells were then sensitized with IgE for 24 h, followed by stimulation with DNP-HSA for 1 h. Western blot analysis was performed. **(D)** RBL2H3 cells were treated with TGF-β1 (5 ng/ml) for 72 h. Cells were then sensitized with IgE for 24 h, followed by stimulation with DNP-HSA for 1 h. The conditioned medium was added to B16F1 cells for 8 h, followed by western blot analysis. **(E)** RBL2H3 cells were transfected with the indicated siRNA. The next day, cells were sensitized with IgE (100 ng/ml) for 24 h, followed by stimulation with DNP-HSA for 1 h. TGF-β1 level was measured. *, p<0.05; ***, p<0.0005. **(F)** RBL2H3 cells were treated with TGF-β1 (5 ng/ml) for 72 h. Cells were then sensitized with IgE for 24 h, followed by stimulation with DNP-HSA for 1 h. The conditioned medium was added to B16F1 cells for invasion or migration potential assays. *, p<0.05; **, p<0.005.

TGF-β1 inhibits the release of histamine from mast cells [24] and suppresses proliferation of mouse mast cells [25] suggesting that TGF-β1 acts as a negative regulator of allergic inflammation. Antigen stimulation decreased the production of TGF-β1 in RBL2H3 cells (Figure 13A). SOCS1-KIR and AG490 restored the production of TGF-β1 in antigen-stimulated RBL2HB3 cells (Figure 13A). TGF-β1 prevented antigen from increasing β-hexosaminidase activity in RBL2H3 cells (Figure 13B) and decreased the expression of FcεRI, JAK2, pJAK2^Y1007^ and SOCS1 in antigen-stimulated RBL2H3 cells (Figure 13C). When added to B16F1 cells, the conditioned medium of antigen-stimulated RBL2H3 cells, increased the expression of SOCS1, HDAC3 and COX-2 (Figure 13D). However, conditioned medium of antigen-stimulated RBL2H3 cells treated with TGF-β1 did not affect the expression of SOCS1, HDAC3 or COX-2 (Figure 13D). The down-regulation of SOCS1 restored the level of TGF-β1 in antigen-stimulated RBL2HB3 cells (Figure 13E). The conditioned medium of antigen-stimulated RBL2H3 cells enhanced the invasion and migration potential of B16F1 cells (Figure 13F). However, the conditioned medium of antigen-stimulated RBL2H3 cells pretreated with TGF-β1 did not enhance the invasion or migration potential of B16F1 cells (Figure 13F). Therefore, SOCS1 and JAK2 mediate allergic inflammation by negatively regulating the production of anti-allergic TGF-β1.

## DISCUSSION

Down-regulation of SOCS1 decreases the expression of HDAC3 (Figure 1B). The role of SOCS1 on the expression of HDAC3 has not been previously reported. HDAC3, through its interaction with rac1, decreases the expression of HDAC2 [20]. HDAC3 binds to the promoter sequences of SOCS1 (Supplementary Figure 1G) and interacts with SOCS1 (Supplementary Figure 1H). The SOCS1 promoter contains binding sites for YY1, SP1, DNMT1, NF-kB, and SNAIL (Personal observations). The role of SNAIL in allergic inflammation has been previously reported [26]. DNMT1 acts as an inhibitor of allergic inflammation [27]. SOCS1 binds to FcεRIβ in antigen-stimulated RBL2H3 cells (Supplementary Figure 1H), suggesting that SOCS1 functions in the FcεRI signaling network in allergic inflammation.

miRNA array analysis shows that miR-122-5p, miR-9-1-5p,-29a-3p and -29c-3p are decreased by antigen in RBL2H3 cells (Figure 4A). TargetScan analysis predicts miR-29a and -29c binding to the 3′-UTR of SOCS1 (Personal observations). It is probable that miR-29a and miR-29c may decrease the expression of SOCS1. miR-9 increases the expression of E-cadherin and suppresses hepatocellular carcinogenesis [28], suggesting that miR-9 inhibits allergic inflammation. miRNA array analysis shows that miR-150, -151 and -196 are increased by antigen in RBL2H3 cells (Figure 4A). These miRNAs may decrease the expression of miRNAs such as miR-122. miR-196 is over-expressed in the cancer tissues and is correlated with lymph node metastasis, promoteing cancer cell migration and invasion through NME4-JNK-TIMP1-MMP signaling [29]. miR-196 may mediate allergic inflammation by increasing the expression of SOCS1. miR-122 enhances interferon signaling by blocking the expression of SOCS1 [30]. miR-122 regulates type I IFN expression through modulating SOCS1 expression [13]. miRNA array analysis shows that miR-122 is decreased by antigen stimulation in RBL2H3 cells (Figure 4A). This led us to hypothesize that miR-122 may acts as a negative regulator of SOCS1. miR-122 decreases luciferase activity associated with SOCS1 (Figure 4E) and the expression of SOCS1 (Figure 4G), which binds to the promoter sequences of miR-122 (Figure 4H). These results indicate that miR-122 and SOCS1 form a negative feedback loop.

Allergic inflammation promotes tumorigenic and metastatic potential [15, 21]. Human mast cells and basophils participate in the complex network involving inflammatory and tumor angiogenesis and lymphangiogenesis [31]. Conditioned medium from LLC (Lewis lung carcinoma) cells increase the expression of cell surface receptors and a pro-angiogenic Runx2/VEGF/Dusp5 axis in mast cells, which promotes tumor angiogenesis [32]. The conditioned medium of antigen-stimulated lung mast cells enhances migration (Figure 5D) and invasion potential (Figure 5E) of B16F1 cells in SOCS1-depedent manner. It would be necessary to identify cytokines that are regulated by SOCS1 for better understanding of interaction between cancer cells and mast cells.

Mast cells and macrophages, activated during allergic inflammation, are necessary for the enhanced metastatic potential of cancer cells that is accompanied by allergic inflammation [18, 20]. Intercellular exosome-mediated miR-122 transfer mediates cellular interaction which is inhibited by IGF-1 [33]. SOCS1 modulates the interaction between tumor and bone marrow accessory cells in multiple myelomas [34]. SOCS1 acts as an inhibitor of IL-4-induced IRS-2 (insulin receptor substrate-2) signaling and anti-inflammatory M2 differentiation [35]. SOCS1 is necessary for cellular interaction involving cancer cells and mast cells (Figure 5) and for cellular interaction involving macrophages and mast cells (Figure 11C). Identification of a novel SOCS1-miRNA network is necessary for better understanding of cellular interactions during allergic inflammation.

SOCS1-KIR inhibits activation of JAK2 and STAT1 in INF-γ-treated explants of human skin [36]. JAK2/STAT3 activation is necessary for macrophage M2 polarization [37]. Allergic inflammation promotes murine macrophage M2 polarization in which the expression of CD163 is increased (Figure 11C). The JAK2-STAT3 axis is responsible for the induction of BMP2, and HDAC3 interacts with STAT3 [38]. HDAC3 knockdown inhibits STAT3 (Tyr705) phosphorylation and survival of pSTAT3-positive DLBCL (Diffuse large B cell lymphoma cells) [39]. These reports suggest the role of JAK2 in allergic inflammation in association with miR-122-SOCS1 feedback loop. SOCS1-KIR binds to JAK2 (Figure 12A) and prevents antigen from increasing phosphorylation of JAK2 (Figure 12B).

Many reports suggest opposing roles of TGF-β1 in allergic inflammation. The activation of human mast cells by FcεRI activates TGF-β1 and leads to cross-talk between mast cells and bronchial epithelial cells [40]. Induced regulatory T cells inhibit mast cell functions through TGF-β1 [41]. Allergen-induced airway remodeling is mediated by AR (aldose reductase) and its inhibition blocks the progression of remodeling via inhibiting TGF-β1-induced PI3K/AKT/GSK3β-dependent pathway [42]. Airway inflammation due to allergic response to RWE, which subsequently activates oxidative stress-induced expression of inflammatory cytokines via a NF-kappaB-dependent mechanism, is prevented by AR inhibitors [43]. These reports imply that TGF-β1 mediates allergic inflammation.

TGF-β1 suppresses FcεRI expression, FcεRI-mediated activation by upregulation of Ehf [44] and inhibits mast cell degranulation [45]. TGF-β1 also inhibits IgE-mediated cytokine production [46]. These reports suggest an anti-allergic function of TGF-β1. Allergic inflammation decreases the production of anti-allergic TGF-β1 in antigen-stimulated RBL2H3 cells (Figure 13A). SOCS1 and JAK2 prevents antigen from decreasing the production of TGF-β1 in antigen-stimulated RBL2H3 cells (Figure 13A). Further study is needed to examine the mechanism of anti-allergic effect of TGF-β1 in association with SOCS1-JAK2 signaling axis. It would be also necessary to further examine the role of TGF-β1 in cellular interactions in tumor microenvironments remodeled by allergic inflammation.

MCP1 is increased in mast cells, macrophages and cancer cells during allergic inflammation (Supplementary Figure 8). Cytokine array analysis revealed that expression of MCP1 was increased by allergic inflammation (data not shown). MCP1, increased during allergic inflammation (Supplementary Figure 3D), mediates cellular interactions among mast cells, macrophages and cancer cells (Supplementary Figure 8). We show that activation of JAK2/STAT signaling occurs in mast cells, macrophages and cancer cell during allergic inflammation. In this study, we present evidence that miR-122-SOCS1-JAK2 signaling axis plays a role for remodeled tumor microenvironment resulting from interaction of cancer cells with stromal cells such as mast cells and macrophages (Supplementary Figure 8).

## MATERIALS AND METHODS

### Materials

Oligonucleotides used in this study were commer-cially synthesized by the Bionex Co. (Seoul, Korea). DNP-HSA, TNP-BSA, DNP-specific IgE antibody and TNP-specific IgE antibody were purchased from Sigma. Chemicals used in this study were purchased from Sigma. All other antibodies were purchased from Cell Signaling Co. (Beverly, MA). Anti-mouse and anti-rabbit IgG-horseradish peroxidase-conjugated antibody was purchased from Pierce. Lipofectamine and PlusTM reagent for transfection were purchased from Invitrogen. SOCS1 mimetic peptide (D-T-H-F-R-T-F-R-S-H-S-D-Y-R-R-I) was commercially synthesized by Peptron Company (Daejon, Korea).

### Cell culture

Rat basophilic leukemia (RBL2H3) cells were obtained from the Korea Cell Line Bank (Seoul, Korea). Cells were grown in Dulbecco's modified Eagle's medium containing heat-inactivated fetal bovine serum, 2 mM L-glutamine, 100 units/ml penicillin, and 100 μg/ml streptomycin (Invitrogen). Cultures were maintained in 5% CO2 at 37°C. The isolation of macrophages from lung tissues was done according to the standard procedures [20]. B16F1 and B16F10 cancer cells used in this study were cultured in Dulbecco's modified minimal essential medium (DMEM; Invitrogen) supplemented with heat-inactivated 10% fetal bovine serum (FBS, Invitrogen) and antibiotics at 37°C in a humidified incubator with a mixture of 95% air and 5% CO2.

### Mice

Five-week-old female BALB/c mice were purchased from Nara Biotech (Seoul, Korea) and maintained in specific pathogen-free conditions. All animal experiments were approved by the institutional review board for animal studies of Kangwon National University. To measure the tumorigenic potential, mouse melanoma B16F1 cells (1 × 10^6^ cells in 100 μl of PBS), after induction of passive systemic anaphylaxis, were injected subcutaneously into the right flank of each mouse (*n* = 5). Tumor growth was evaluated by measuring the tumor diameters with calipers and calculating the tumor volumes using an approximated formula for a prolate ellipsoid as follows: volume = ((*a*.*b*2)/2), where *a* is the longest axis of the tumor, and *b* is the shortest axis. To determine the effect of SOCS1 on the enhanced tumorigenic potential by allergic inflammation, scrambled siRNA (100 nM) or SiSOCS1 RNA (100 nM) was injected intravenously five times, after B16F1 cell injection in a total of 24 days. To determine the effect of SOCS1 mimetic peptide on the enhanced tumorigenic potential by allergic inflammation, SOCS1 mimetic peptide was injected intravenously ten times, after B16F1 cell injection in a total of 42 days.

### Isolation of lung mast cells

Lung tissues of BALB/C mice were cut into fragments and incubated in modified Tyrode's buffer (137 mmol/L NaCl, 2.8 mmol/L KCl, 12 mmol/L NaHCO3, 0.49mmol/L MgCl2, 0.4 mmol/L NaH2PO4, 5.5mmol/L glucose, 10mmol/L HEPES, 3.5 mg/ml BSA) medium supplemented with 0.5 mg/ml collagenase (Sigma-Aldrich, St. Louis, MO) for 15 min at 37°C. The supernatant tissue pellets were collected and resuspended with collagenase solution in Tyrode's buffer at 37°C for another 30 min. After 30 min, 10 ml of 0.015 mg/ml DNase I (Sigma-aldrich, St. Louis, MO) solution in PBS was added to tissue pellet and incubated at 37°C for another 30 min. To remove the large undigested tissue pieces, the tissue pellets were filtered through 70 μm strainers (Becton Dickinson Labware, Franklin Lakes, New Jersey). The pelleted cells were resuspended in lysis buffer (1.37 g NH4Cl, 0.515 g Tris, 250 ml ddH20, pH7.2) to remove RBC and incubated for 5 min at RT. After addition of 10 ml PBS/DNase solution to stop the lysis reaction and centrifugation 200g for 10 min, the cell pellets were resuspended in DNase I solution in RPMI media and subjected to a continuous isotonic Percoll gradient (72%). Purified mast cells were resuspended in RPMI-FBS. The cell purity (> 96%) and viability (> 98%) were evaluated by toluidine blue and trypan blue exclusion staining, respectively.

### Monitoring of rectal temperature

Changes in core body temperature associated with systemic anaphylaxis were monitored by measuring changes in rectal temperatures using a rectal probe coupled to a digital thermometer.

### β-Hexosaminidase activity assays

The β-hexosaminidase activity assay was performed according to standard procedures [21].

### Western blot analysis

Western blot and immunoprecipitation were performed according to the standard procedures [22].

### Chromatin immunoprecipitation (ChIP) assay

Assays were performed according to the manu-facturer's instruction (Upstate). The HDAC3 antibody immunoprecipitates were reverse cross-linked. PCR was done on the phenol/chloroform-extracted DNA with specific primers of the SOCS1 promoter-1 (5′- CGGAGCCCTAACCAGAAGAA-3′ (sense) and 5′- TCTTAAACCAGGCAGGCCC-3′ (antisense)), SOCS1 promoter-2 (5′-TTGCCGGAAAGAGAAACCGA -3′ (sense) and 5′-CATCCTCGACCCTGCCATAC-3′ (antisense)), and SOCS1 promoter-3 (5′-GTATGGCAGGGTCGAGGATG-3′ (sense) and 5′-GTGGGCTCATCTGCGAAGTA-3′ (antisense)) sequences were used to examine the binding of HDAC3 to the SOCS1 promoter sequences. miR-122 promoter-1 (5′- TCCCCCTTCTCAACATTTGG-3′ (sense) and 5′- GCAGGTGAGGGGTCCAACTA -3′ (antisense)), miR-122 promoter-2 (5′- CTGCTGCCTGCAGTTCTTCT-3′ (sense) and 5′- CCCTTCCCTTCCTTTCCTTT-3′ (antisense)), and miR-122 promoter-3 (5′- GAAAGGAAAGGAAAGGAAAGGA-3′ (sense) and 5′- TCCAAACGCTGAAACTGGAG -3′ (antisense)) were used to examine the binding of protein of interest to the miR-122 promoter sequences.

### miR-122 and pGL3-3′UTR-SOCS1 construct

To generate miR-122 expression vector, 216 bp genomic fragment encompassing primary miR-122 gene was PCR amplified and cloned into (GGATCC/CTCGAG) site of pcDNA3.1 vector. To generate pGL3-3′UTR-SOCS1 construct, 342 bp mouse SOCS1gene segment encompassing 3′UTR was PCR amplified and subcloned into (TCTAGA/TCTAGA) site of pGL3 luciferase plasmid. Luciferase activity assay was performed according to the instruction manual (Promega Company).

### Immunofluorescence staining

RBL2H3 cells were seeded onto glass coverslips in 24-well plates and were sensitized with IgE (100 ng/ml) for 16 h. After stimulation with DNP-HSA (100 ng/ml) for 1 h, cells were fixed with 4% paraformaldehyde (v/v) for 10 min and then permeabilized with 0.4% Triton X-100 for 10 min. Nonspecific antibody-binding sites were blocked by incubation with 1% BSA in TBST for 30 min. Cells were then incubated with primary antibody specific to HDAC3 (1:100; Santa Cruz Biotechnology), SOCS1 (1:100; Santa Cruz Biotechnology), CD163 (1:100; Santa Cruz Biotechnology) or FcεRIβ (1:100; Santa Cruz Biotechnology) for 2 h, followed by washing with TBS-T three times. Anti-goat IgG-FITC (for detection of HDAC3 and SOCS1) or anti-rabbit Alexa Fluor 586 (for detection of FcεRIβ and CD163) secondary antibody (Molecular Probes) was added to cells and incubated for 1 h. Coverslips were then washed and mounted by applying Mount solution (Biomeda, Foster City, CA). Fluorescence images were acquired using a confocal laser scanning microscope and software (Fluoview version 2.0) with a X 60 objective (Olympus FV300, Tokyo, Japan). To examine the effect of SOCS1 on the co-localization of HDAC3 with FcεRIβ, RBL2H3 cells were transfected with scrambled siRNA (10 nM) or SOCS1 siRNA (10 nM). The next day, cells were sensitized with IgE (100 ng/ml) for 24 h, followed by stimulation with DNP-HSA (100 ng/ml) for 1 h. Immunofluorescence staining was performed.

### miRNA target analysis

Genes that contain the miR-binding site(s) in the UTR were obtained using the TargetScan program (http://www.targetscan.org/, http://pictar.mdc-berlin.de/, http://www.microrna.org/microrna/home.do).

### RNA Extraction and Quantitative Real Time PCR

Total miRNA was isolated using the *mir*VanamiRNA isolation kit (Ambion). miRNA was extended by a poly(A) tailing reaction using the A-Plus poly(A) polymerase tailing kit (Cell Script). cDNA was synthesized from miRNA with poly(A) tail using a poly(T) adaptor primer and qScriptTM reverse transcriptase (Quanta Biogenesis). Expression levels of miR-384 or miR-122 was quantified with SYBR Green quantitative RT-PCR kit (Ambion) using an miRNA-specific forward primer and a universal poly (T) adaptor reverse primer. The expression of miR-122 was defined based on the threshold (*Ct*), and relative expression levels were calculated as 2^−(*Ct* of miR-122)-(*Ct* of U6)^ after normalization with reference to expression of U6 small nuclear RNA. For quantitative PCR, SYBR PCR Master Mix (Applied Biosystems) was used in a CFX96 Real Time System thermocycler (Bio-Rad).

### Transfection

Transfections were performed according to the manufacturer's instructions. Lipofectamine and Plus reagents (Invitrogen) were used. For miR-122 knockdown, cells were transfected with 10 nM oligonucleotide (inhibitor) with Lipofectamine 2000 (Invitrogen), according to the manufacturer's protocol. The sequences used were 5′- CAAACACCAUUGUCACACUCCA -3′ (miR-122 inhibitor) and 5′- UUCUCCGAACGUGUCACGUTT -3′ (control inhibitor).

### Passive cutaneous anaphylaxis

BALB/C mice were sensitized with an intradermal injection of IgE (0.5 μg/kg). Twenty four hours later, mice were challenged with an intravenous injection of DNP-HSA (250 μg/kg) and 2% (v/v) Evans blue solution. Thirty minutes after DNP-HSA challenge, the mice were euthanized, and the 2% (v/v) Evans blue dye was extracted from each dissected ear in 700 μl of acetone/water (7:3) overnight. The absorbance of Evans blue in the extracts was measured with a spectrophotometer at 620 nm. To determine the effect of SOCS1 on the PCA, BALB/C mice were given an intravenous injection of scrambled siRNA (100 nM) or SiSOCS1 RNA (100 nM) on the next day of the sensitization with IgE. One hour after the injection of siRNA, BALB/C mice were challenged with DNP-HSA (250 μg/kg) and 2% (v/v) evans blue solution for determining the extent of vascular permeability accompanied by PCA.

### Effect of passive systemic anaphylaxis on tumorigenic potential

For induction of passive systemic anaphylaxis, BALB/c mice were sensitized by intravenous injection of IgE (0.5 μg/kg). The next day, sensitized mice were intravenously injected with DNP-HSA (250 μg/kg). Two days after injection of DNP-HSA, B16F1 mouse melanoma cells (1 × 10^6^ cells) were injected into the flanks of each BLAB/c mouse to examine the effect of systemic anaphylaxis on tumorigenic potential. To determine the effect of SOCS1 on the enhanced tumorigenic potential by PSA, scrambled siRNA (100 nM) or SOCS1 siRNA (100 nM) was injected intravenously into BALB/c mice three days after B16F1 cell injection. To determine the effect of PSA on the metastatic potential of mouse melanoma cells, BALB/c mice were injected intravenously with IgE (0.5 μg/kg). The next day, BALB/c mice were injected intravenously with DNP-HSA (250 μg/kg). Two days after injection of DNP-HSA, mouse melanoma B16F1 cells (1 × 10^6^ cells) were intravenously injected into BALB/c mice.

### Matrigel plug assays

Seven week-old BALB/C mice (DBL Co., Ltd, Korea) were injected subcutaneously with 0.1 ml of matrigel containing the conditioned medium and 10 units of heparin (Sigma). The injected matrigel rapidly formed a single, solid gel plug. After 8 days, the skin of the mouse was easily pulled back to expose the matrigel plug, which remained intact. Hemoglobin (Hb) content in the matrigel plugs was measured using the Drabkin reagent (Sigma, USA) for quantification of blood vessel formation.

### Effect of passive systemic anaphylaxis on metastatic potential

For induction of passive systemic anaphylaxis, BALB/C mice were sensitized by an intravenous injection of IgE (0.5 μg/kg). The next day, sensitized mice were challenged by an intravenous injection of DNP-HSA (250 μg/kg). Three days after the injection of IgE, BALB/C mice were given an intravenous injection of B16F1 melanoma cells (2 X10^5^). To determine the effect of SOCS1 on the enhanced metastatic potential of cancer cells by PSA, BALB/C mice were given an intravenous injection with scrambled (100 nM) or SOCS1 siRNA (100 nM) on days 1, 7, and 10 of the time line. On day 14 of the time line, lung tumor tissues were harvested. The extent of metastasis was determined as described. To determine the effect of SOCS1 mimetic peptide on the metastatic potential of cancer cells, BALB/C mice were given an intravenous injection with SOCS1 mimetic peptide (9 mg/kg) on days 7, 10, and 14 of the time line. On day 17 of the time line, lung tumor tissues were harvested.

### Wound migration assays

Cells were plated overnight to achieve a confluent layer in 24-well plates. A scratch was made on the cell layer with a micropipette tip, and cultures were washed twice with serum-free medium. Wound healing was visualized by comparing photographs taken at the time of 0 and 48 h post scratch. To examine the effect of mast cells on the migration potential of B16F1 melanoma cells, the conditioned medium of lung mast cells obtained after PSA was added to B16F1 melanoma cells in serum-free medium in a 1:1 ratio.

### Chemo invasion assays

The invasive potential was determined by using a transwell chamber system with 8 -μm pore polycarbonate filter inserts (CoSTAR, Acton, MA). The lower and upper sides of the filter were coated with gelatin and Matrigel gel, respectively. Trypsinized cells (5 × 10^3^) in serum-free RPMI 1640 medium containing 0.1% bovine serum albumin were added to each upper chamber of the transwell. RPMI 1640 medium supplemented with 10% fetal bovine serum was placed in the lower chamber, and cells were incubated at 37°C for 16 h. The cells were fixed with methanol, and the invaded cells were stained and counted. Results were analyzed for statistical significance using the Student's *t* test. Differences were considered significant when p < 0.05.

### Immunohistochemical staining

Immunohistochemical staining of lung tissues was also performed using an established avidin-biotin detection method (Vectastain ABC kit, Vector Laboratories Inc., Burlingame, CA). Briefly, 4-6 μm-thick sections of the paraffin-embedded tissue blocks were cut, mounted on positively charged glass slides, and dried in an oven at 56°C for 30 min. The sections were deparaffinized in xylene and then rehydrated in graded ethanol and water. Endogenous peroxidase was blocked by incubation in 3% (v/v) hydrogen peroxide for 15 min. Antigen retrieval was accomplished by pretreatment of the sections with citrate buffer at pH 6.0 for 20 min at 56°C in a microwave oven and then allowing the sections to cool for 30 min. Nonspecific endogenous protein binding was blocked using 1% bovine serum albumin (BSA). The sections were then incubated with primary antibodies overnight at 4°C. The following primary antibodies were used for single and double staining: anti-SOCS1 (1:100, Santa Cruz Biotechnology), anti-pJAK2^Y1007^ (1:100, Santa Cruz Biotechnology). After washing, biotinylated secondary antibodies were applied at 1:100 or 1:200 dilutions for 1 h. Color was developed with diaminobenzidine (Vector Laboratories, Inc.). Sections were counterstained with Mayer's hematoxylin. Sections incubated without primary antibody served as controls. To visualize tissue mast cells, the sections were stained with 0.1% olivine blue (Sigma) in 0.1 N HCl for 15 min.

### Assay of TGF-β1 release

TGF-β1 secretion was measured according to manufacturer's instructions (AbCam). Briefly, after washing the 96-well plates coated with monoclonal antibody to mouse TGF-β1, cell culture supernatants were loaded on plate and incubated at room temperature for 2 hours on a shaker at 400 rpm. After washing five times with wash buffer, biotin-conjugated anti-mouse TGF-β1 antibody was applied, followed by Streptavidin-HRP and finally the 3,3′,5,5′-Tetramethylbenzidine substrate. The plates were read using a microplate reader at 450 nm. To detect the effect of SOSC1 and JAK2 on TGF-β1 secretion, SOSC1 KIR (5 μM) and AZ490 (0.1 μM) were pretreated respectively for 3 hours before DNP-HSA stimulation.

### Statistical analysis

Data were analyzed and graphed using GraphPad Prism statistics program (GraphPad Prism software). Results are presented as means± S.E. Statistical analysis was performed using *t* tests with differences between means considered significant when *p* < 0.05.

## SUPPLEMENTARY MATERIALS FIGURES


