# Supplementary material for: miR-122-SOCS1-JAK2 axis regulates allergic inflammation and allergic inflammation-promoted cellular interactions
Source: Oncotarget. 2017 Jul 10;8(38):63155–76. doi: 10.18632/oncotarget.19149 (PMC5609911; doi:10.18632/oncotarget.19149)
Supplement: Supplementary file 1 [file oncotarget-08-63155-s001.pdf]

miR-122-SOCS1-JAK2 axis regulates allergic inflammation and allergic inflammation-promoted cellular interactions

SUPPLEMENTARY MATERIALS

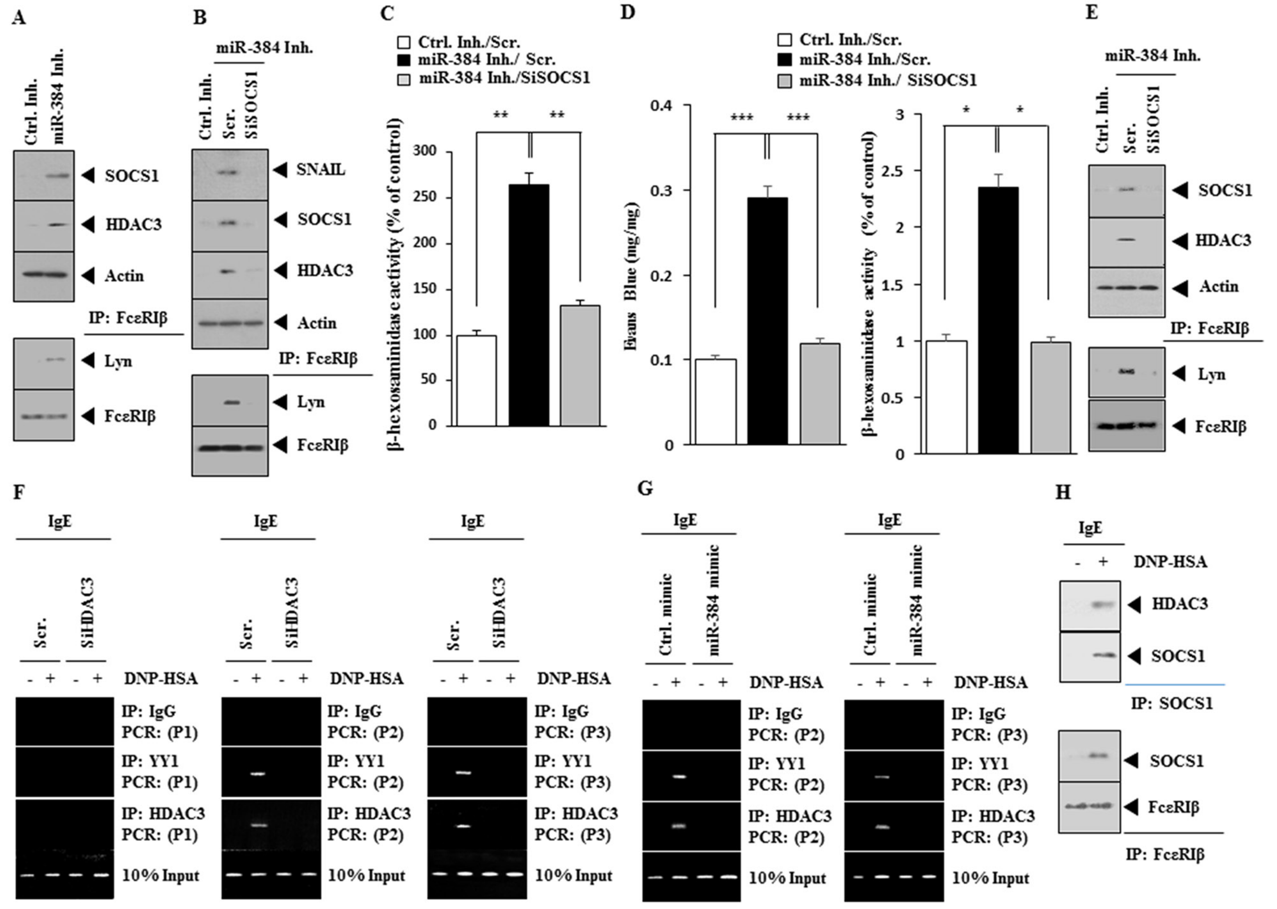

Supplementary Figure 1: miR-384-HDAC3 feedback loop regulates the expression of SOCS1and allergic inflammation. **(A)** RBL2H3 cells were transfected with the indicated inhibitor (each at 10 nM), followed by western blot and immunoprecipitation. **(B)** RBL2H3 cells were transfected with the indicated inhibitor along with scrambled siRNA (10 nM) or SOCS1 siRNA (10 nM). **(C)** Same as (B) except that β-hexosaminidase activity assays were performed. **, $p<0.005$. **(D)** BALB/c mice were given an intravenous injection of scrambled (100 nM) or SOCS1 siRNA (100 nM) along with the indicated inhibitor. The next day, BALB/c mice were given an intravenous injection of 2% (v/v) Evans blue solution (left panel). Ear tissue lysates from BALB/c mouse of each experimental group were subjected to β-hexosaminidase activity assay (right panel). *, $p<0.05$; ***, $p<0.0005$. **(E)** Ear tissue lysates were subjected to western blot and immunoprecipitation. **(F)** RBL2H3 cells were transfected with scrambled siRNA or HDAC3 siRNA. The next day, cells were sensitized with IgE for 24 h, followed by stimulation with DNP-HSA. One hour after stimulation with antigen, ChIP assays were performed. P1, P2 and P3 denote region of SOCS1 promoter amplified by primer 1, 2 and 3, respectively. **(G)** Same as (D) except that RBL2H3 cells were transfected with control mimic (10 nM) or miR-384 mimic (10 nM). P1, P2 and P3 denote region of SOCS1 promoter amplified by primer 1, 2 and 3, respectively. **(H)** The IgE-sensitized RBL2H3 cells were stimulated with DNP-HSA for 1 h, followed by western blot and immunoprecipitation.

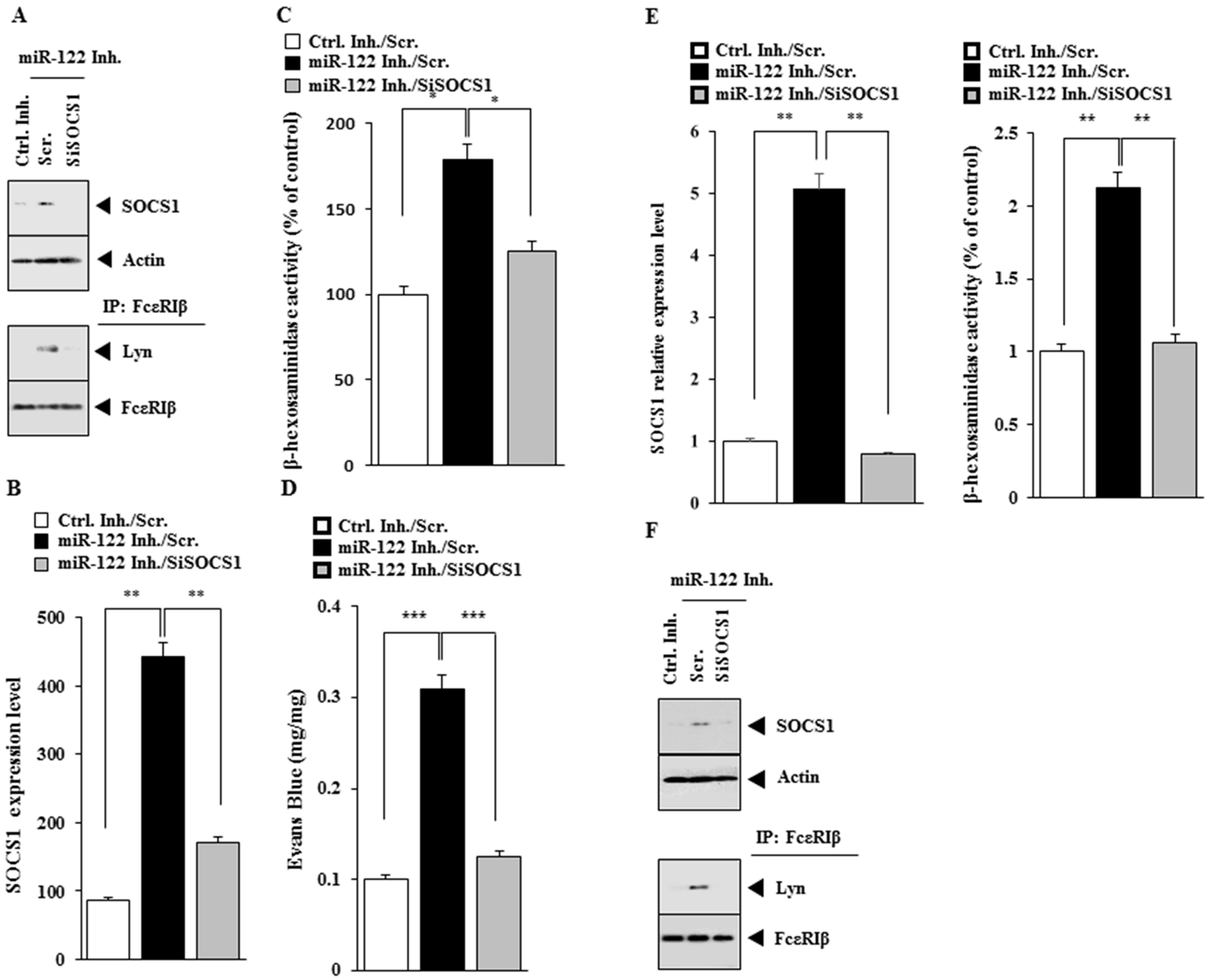

Supplementary Figure 2: miR-122 inhibits *in vivo*features of allergic inflammation. **(A)** RBL2H3 cells were transfected with the indicated inhibitor along with scrambled siRNA or SOCS1 (each 10 nM). Immunoprecipitation and western blot were performed. **(B)** Same as (A) except that qRT-PCR analysis was performed. **, p<0.005. **(C)** Same as (B) except that β-hexosamindase activity assays were performed. *, p<0.05. **(D)** BALB/c mice were given an intravenous injection of scrambled (100 nM) or SOCS1 siRNA (100 nM) along with the indicated inhibitor (each at 100 nM)). The next day, BALB/c mice were given an intravenous injection of 2% (v/v) Evans blue solution. ***, p<0.0005. **(E)** Ear tissue lysates from BALB/c mouse of each experimental group were subjected to qRT-PCR analysis and β-hexosaminidase activity assay. **, p<0.005. **(F)** Ear tissue lysates from BALB/c mouse of each experimental group were subjected to western blot and immunoprecipitation.

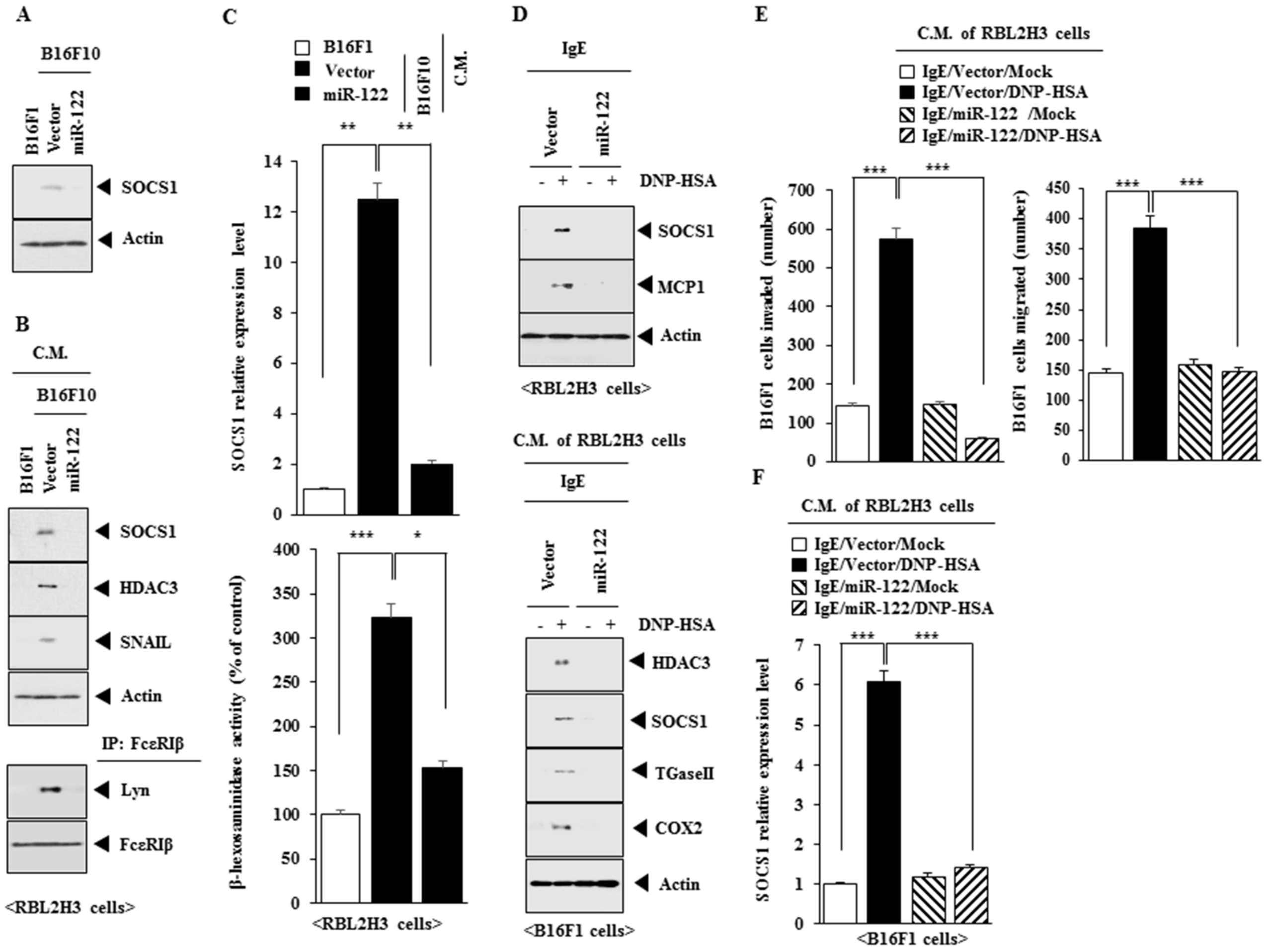

Supplementary Figure 3: miR-122 inhibits the effects of cancer cells on allergic inflammation. **(A)** Western blot was performed. **(B)** At 48 h after transfection, conditioned medium was added to RBL2H3 cells for 8 h, followed by western blot. **(C)** Same as (B) except that qRT-PCR and β-hexosaminidase activity assays were performed. *, $p<0.05$; **, $p<0.005$; ***, $p<0.0005$. **(D)** RBL2H3 cells were transfected with the indicated construct. The next day, cells were sensitized with IgE for 24 h, followed by stimulation with DNP-HSA, followed by western blot (upper panel). One hour after stimulation with DNP-HSA, conditioned medium was obtained and added to B16F1 cells for 8 h, followed by western blot (lower panel). **(E)** The conditioned medium from RBL2H3 cells was added to B16F1 cells, followed by chemoinvasion or wound migration assays. ***, $p<0.0005$. **(F)** Same as (D) (lower panel) except that qRT-PCR analysis was performed. ***, $p<0.0005$.

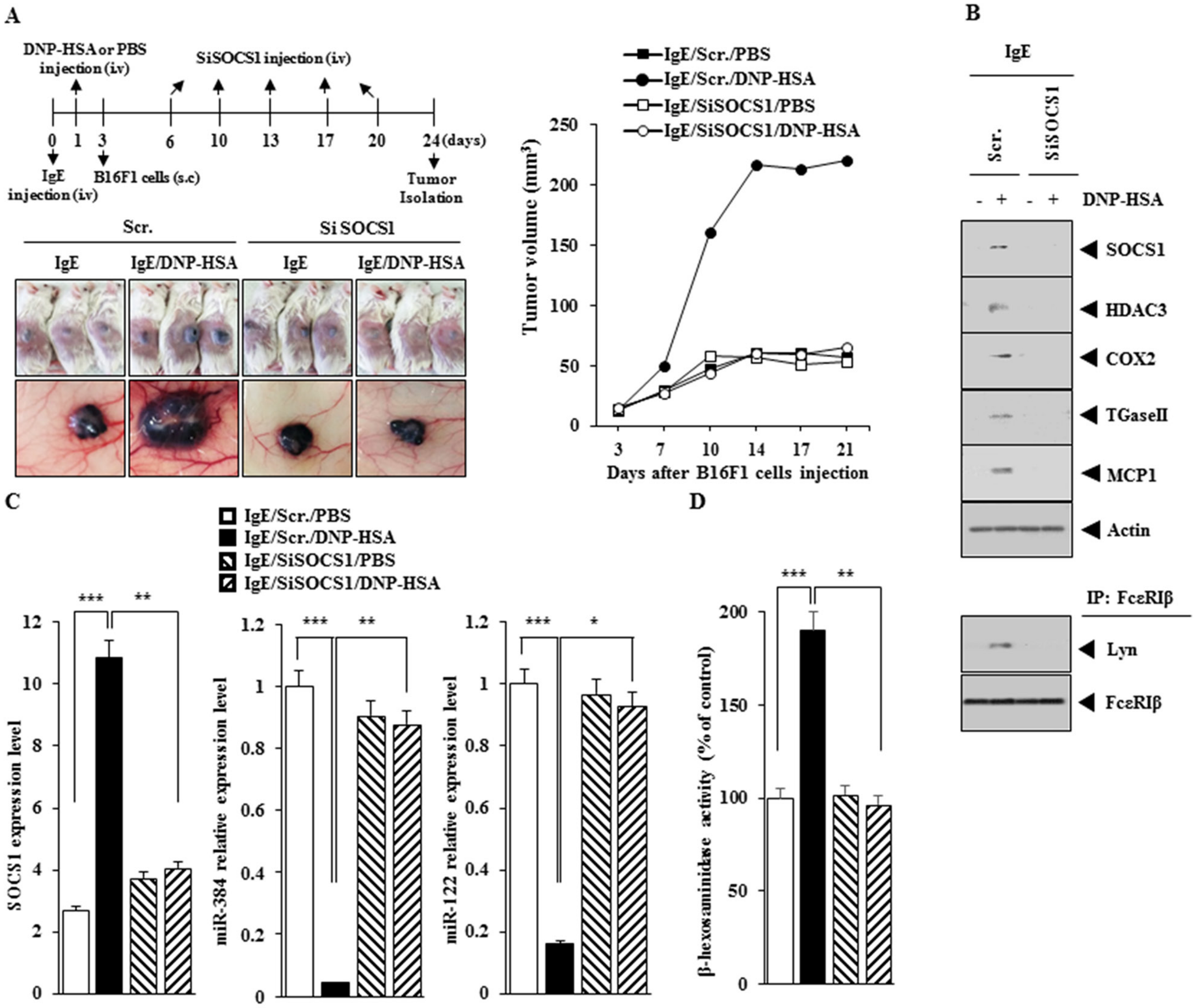

Supplementary Figure 4: SOCS is necessary for the enhanced tumorigenic potential of B16F1 cells by allergic inflammation. **(A)** BALB/C mice were sensitized to IgE (0.5 μg/kg) by an intravenous injection. The next day, BALB/C mice were given an intravenous injection of DNP-HSA (250 μg/kg). Each mouse received injection of B16F1 melanoma cells (2X10^5) on the day 3 of the time line. After tumor reached a certain size, BALB/C mice were given an intravenous injection with scrambled siRNA (100 nM) or SiSOCS1 RNA (100 nM) on days 6, 10, 13, 17, and 20 of the time line. Twenty one days after the injection of B16F1 cells, the tumorigenic potential of B16F1 cells was determined. **(B)** Tumor tissue lysates from each experimental group were subjected to western blot and immunoprecipitation. **(C)** qRT-PCR analysis of tumor tissue lysates was performed. *, $p<0.05$; **, $p<0.005$; ***, $p<0.0005$. **(D)** β-hexosaminidase activity assays were performed. **, $p<0.005$; ***, $p<0.0005$.

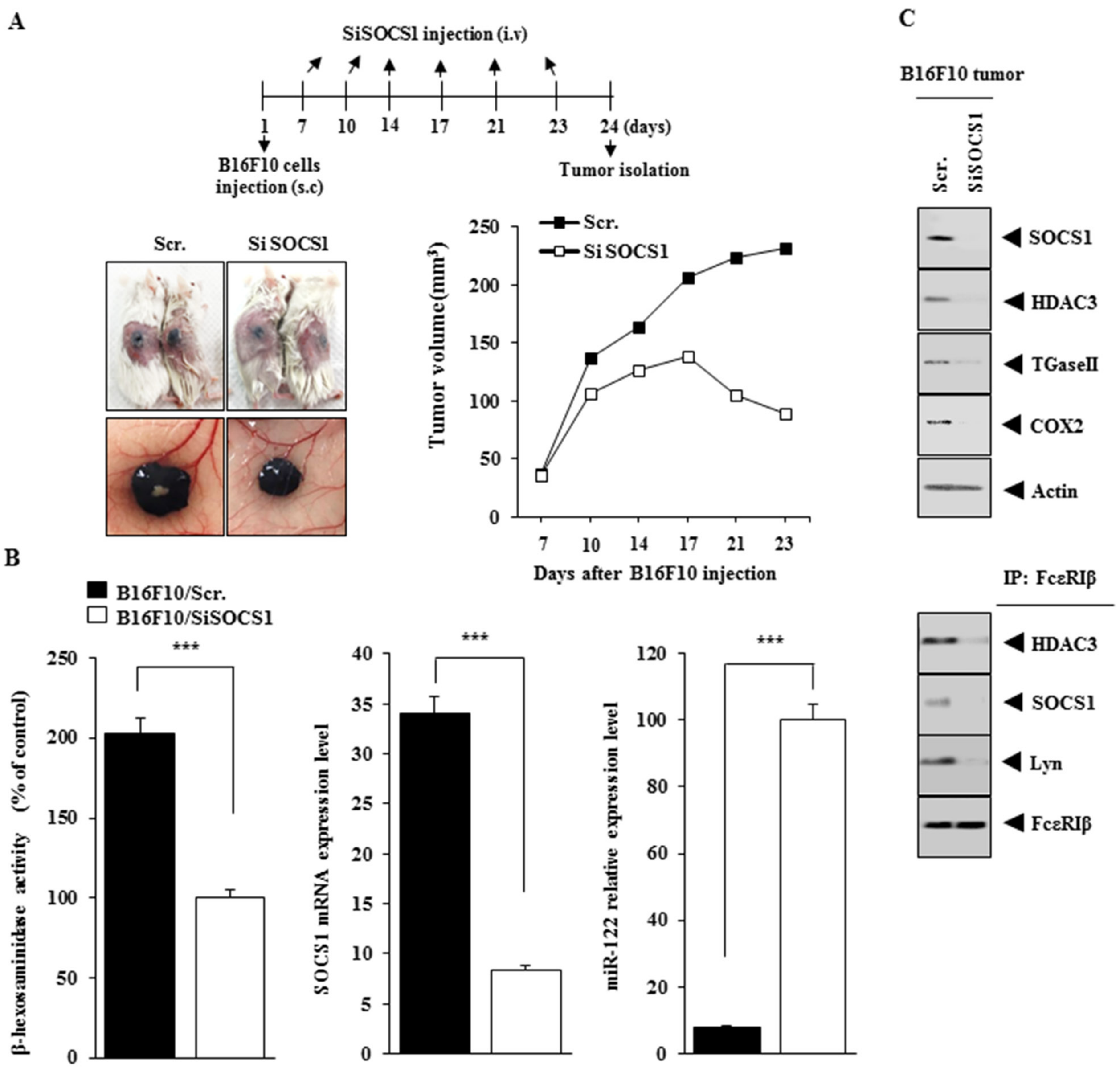

Supplementary Figure 5: SOCS1 is necessary for the tumorigenic potential of B16F10 melanoma cells and the cellular interaction. **(A)** BALB/C mice were given a subcutaneous injection of B16F10 melanoma cells (2X10^5). BALB/C mice were given an intravenous injection with scrambled siRNA (100 nM) or SiSOCS1 RNA (100 nM) on days 7, 10, 14, 17, 21and 23 of the time line. **(B)** Tumor tissue lysates were subjected to β-hexosaminidase activity assays and qRT-PCR analysis. ***, $p<0.0005$. **(C)** Tumor tissue lysates from each experimental group were subjected to western blot and immunoprecipitation.

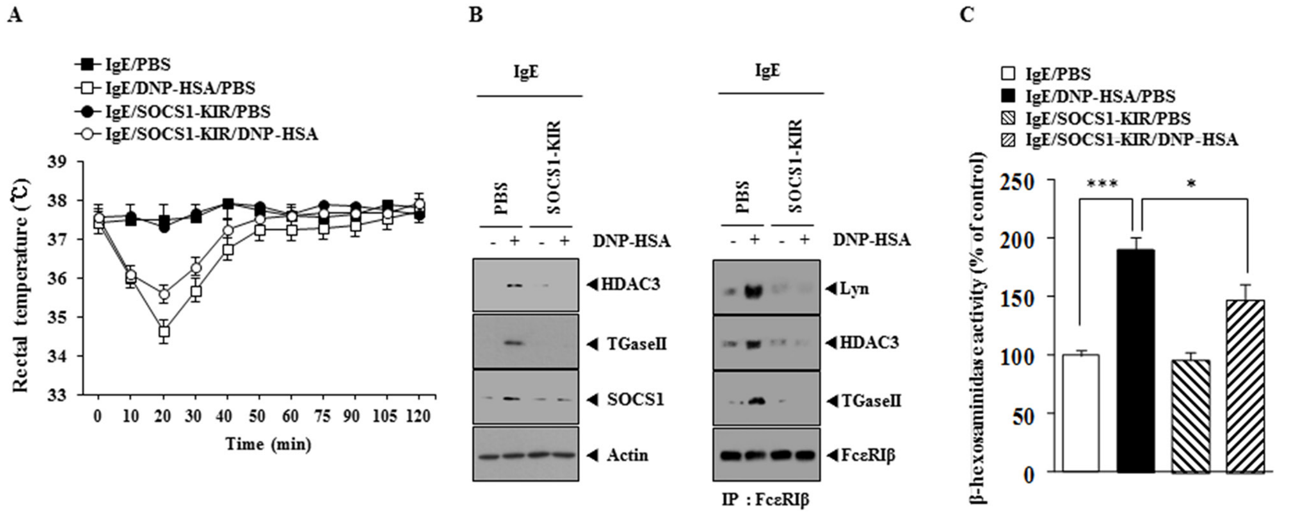

Supplementary Figure 6: SOCS1-KIR mimetic peptide inhibits PSA. **(A)** BALB/C mice were sensitized to IgE (0.5 μg/kg) by an intravenous injection into the tail vein. The next day, BALB/C mice were given an intravenous injection of DNP-HSA (250 μg/kg) along with SOCS1-KIR (9 mg/kg). At each time point, rectal temperature was measured. **(B)** One hour after stimulation with DNP-HSA, lung tissue lysates from each mouse of each experimental group of mice were subjected to western blot and immunoprecipitation. **(C)** Same as (B) except that β-hexosaminidase activity assays were performed. *, $p<0.05$; ***, $p<0.0005$.

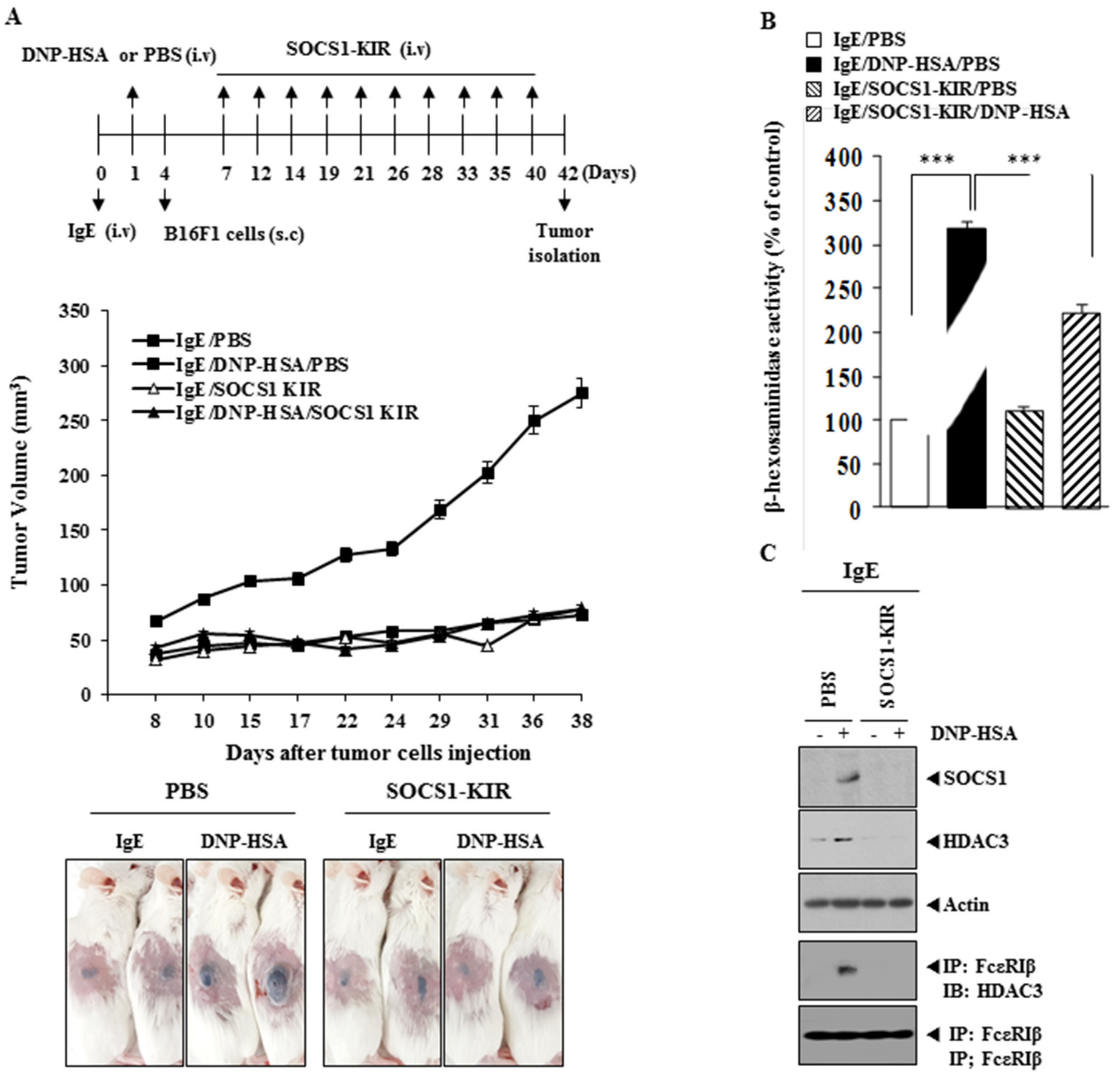

Supplementary Figure 7: SOCS1-KIR mimetic peptide inhibits allergic inflammation-promoted enhanced tumorigenic potential of B16F1 melanoma cells. **(A)** BALB/C mice were sensitized with IgE (0.5 μg/kg) by an intravenous injection. The next day, BALB/C mice were given an intravenous injection of DNP-HSA (250 μg/kg). Each mouse received intravenous injection of B16F1 melanoma cells (2X10^5) on the day 4 of the time line and received intravenous injection of SOCS1-KIR (9 mg/kg) at the indicated day of the time line. On the day 42 of the time line, tumor tissues were harvested. **(B)** Tumor tissue lysates from each mouse of each experimental group of mice were subjected to β-hexosaminidase activity assays. ***, p<0.0005. **(C)** Tumor tissue lysates from each mouse of each experimental group of mice and were subjected to western blot and immunoprecipitation.

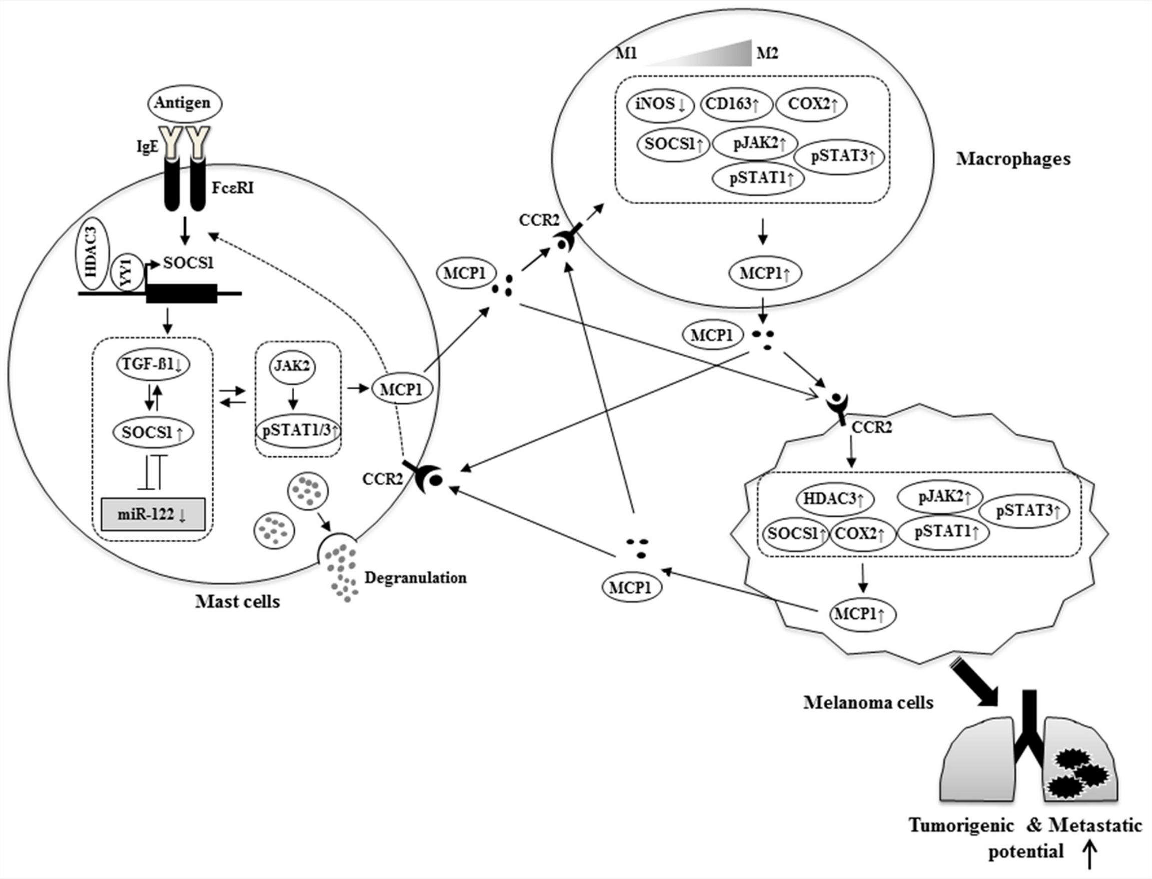

Supplementary Figure 8: Regulatory role of miR-122-SOCS1-JAK2 loop in allergic inflammation and the cellular interactions promoted by allergic inflammation.
